# Breast cancer pathogenesis, diagnosis and treatment: a comprehensive review

**DOI:** 10.3389/fonc.2026.1773232

**Published:** 2026-03-26

**Authors:** Darshika Pasi, Bijulal Aswathy, Mrinmoy Das, Piyush Agrawal

**Affiliations:** 1 Department of Biotechnology, St. Aloysius College, Jabalpur, Madhya Pradesh, India; 2 Division of Medical Research, SRM Medical College Hospital and Research Centre, Faculty of Medicine and Health Sciences, SRM Institute of Science and Technology, Kattankulathur, Chengalpattu, Tamilnadu, India

**Keywords:** artificial intelligence, breast cancer, chemotherapy, estrogen, human epidermal growth factor receptor 2, immunotherapy, luminal, pathogenesis

## Abstract

**Background:**

Breast cancer continues to be the leading global health concern and cause of cancer-related deaths among women, with about 20 million new cases and 9.7 million deaths estimated globally based on GLOBOCAN 2022 data. Breast cancer exhibits unique epidemiological patterns and heterogeneity that influence several characteristic features including tumor development, metastatic potential, treatment response, and outcomes. The knowledge of the complex interplay between genetic, environmental, and microenvironmental factors is critical for developing better diagnostic and therapeutic approaches.

**Methods:**

A comprehensive literature search was carried out to review and compile the progress made in the field of breast cancer epidemiology, molecular subtypes, pathogenesis, diagnostic tools, and treatment strategies. The focus was on genetic susceptibility, hormonal and behavioral risk factors, tumor microenvironment, novel biomarkers, novel imaging and liquid biopsy techniques, and novel treatment strategies, besides recent advances in bioinformatics and AI-based analyses.

**Results:**

Breast cancer can be broadly classified into major molecular subtypes i.e., triple-negative breast cancers, Luminal A, Luminal B, and HER2-enriched. Each subtype exhibits unique biological behavior and therapeutic vulnerabilities. Tumor growth is driven by intricate interactions between the tumor microenvironment (TME) and immune cells, inflammatory mediators, extracellular matrix remodeling, hypoxia, cancer stemness, cellular senescence, and metabolic dysregulation. Progress in diagnostics have been made, with the integration of molecular characterization, genomics, and non-invasive liquid biopsies, in addition to traditional imaging and histopathology. Therapeutic modalities have expanded beyond conventional approaches such as surgery, chemotherapy, and radiation to modern day approaches such as targeted therapies, antibody-drug conjugates, immunotherapy, plant and nanomedicine-based therapies, and new cellular therapies. Also, computational biology and artificial intelligence (AI)-based approaches are now rapidly increasing for biomarker identification, treatment decision-making, and patient outcome prediction.

**Conclusion:**

In summary, the current review provides an updated and comprehensive prospective in complex nature of breast cancer development, diagnostic approaches, and treatment options.

## 1 Introduction

Breast cancer remains to be the frequently diagnosed malignancy among women worldwide. Breast cancer is one of the leading global health concerns and cause of cancer-related deaths among women, with about 20 million new cases and 9.7 million deaths estimated globally based on GLOBOCAN 2022 data (1, 2). Epidemiological studies have reported that approximately one out of seven women will develop breast cancer during their lifetime, underscoring its significant public health burden and long-term clinical impact (3). Over the past two to three decades, substantial advancements have been made in early detection, understanding the molecular basis of the disease, and personalized treatment for breast cancer. As per research, 70-80% of breast cancer has a higher chance of being cured when non-metastatic breast cancer is diagnosed at an early stage, despite the presence of various inequities in treatment and accessibility all over the world. However, when breast cancer is at an advanced stage and has already metastasized, it is still very far from being cured by the current strategies being used (4). A complex interaction between genetic, hormonal, lifestyle, and environmental factors shape the burden of breast cancer. It is well known fact that inherited mutations in genes like *TP53, PTEN, BRCA1*, and *BRCA2* increase susceptibility (5). Hormones, especially progesterone and estrogen, also play a crucial role in the development of tumor and its metastasis. Lifestyle factors such as smoking, alcohol use, obesity, and physical inactivity further contribute and increase the risk of having breast cancer. Furthermore, environmental exposures like radiation and chemicals that disrupt hormones have also been linked in recent studies (6).

A deeper and correct understanding of breast cancer molecular classification is essential for accurate diagnosis and effective treatment. Based on the presence of three different receptors, i.e., estrogen receptor (ER), progesterone receptor (PR), and human epidermal growth factor receptor 2 (HER2), breast cancer is broadly categorized into 4 subtypes, i.e., basal/triple negative, HER2, luminal A, and luminal B. These four subtypes also differ in prognosis and therapeutic responses (7). Breast cancer pathogenesis is regulated by several factors including genetic mutations (8), epigenomic changes (9), and dynamic interactions happening within the tumor microenvironment (TME) (10). Immune components, including macrophages, tumor-infiltrating lymphocytes (TILs), enzymes such as matrix metalloproteinases (MMPs), lysyl oxidases (LOX), vascular endothelial growth factor (VEGF), hypoxia inducible factor-1a (HIF-1α), etc., also play pivotal roles in tumor progression and immune evasion (11–13).

Breast cancer can be diagnosed through both conventional and contemporary methods such as mammography, magnetic resonance imaging (MRI), tissue biopsy, immunohistochemistry (IHC), and gene expression by next-generation sequencing (NGS) technology (14). In addition, advances made in the liquid biopsy techniques, such as exosomal miRNAs (exomiRs), circulating tumor DNA (ctDNA), etc., open a new avenue and offer less invasive and real-time monitoring facilities (15). In addition to systemic treatments like chemotherapy and hormone therapy, therapeutic approaches include surgery, radiotherapy, and targeted agents like trastuzumab and CDK4/6 inhibitors (16). Likewise, the advent of immunotherapy, particularly immune checkpoint inhibitors, i.e., anti-PD1, anti-CTLA4, etc., has created new treatment options to treat complex subtypes like triple-negative breast cancer (TNBC) (17, 18).

Recent advancements in bioinformatics methods, artificial intelligence (AI) and machine learning (ML) have transformed the area of breast cancer studies (19). These tools perform high-throughput sequencing analysis of multiple omics data types (transcriptome, epigenome, proteome, and metabolome), single-cell data, gene enrichment analysis such as pathway mapping, in silico drug discovery, and predictive modeling (20, 21). Deep learning (DL) models now accurately interpret mammograms, H&E slides, and clinical data analysis using natural language processing (NLP) (22, 23). Such analysis has led to enhanced accuracy and efficiency in breast cancer diagnosis, prognosis, and therapeutics.

**Figure 1:**
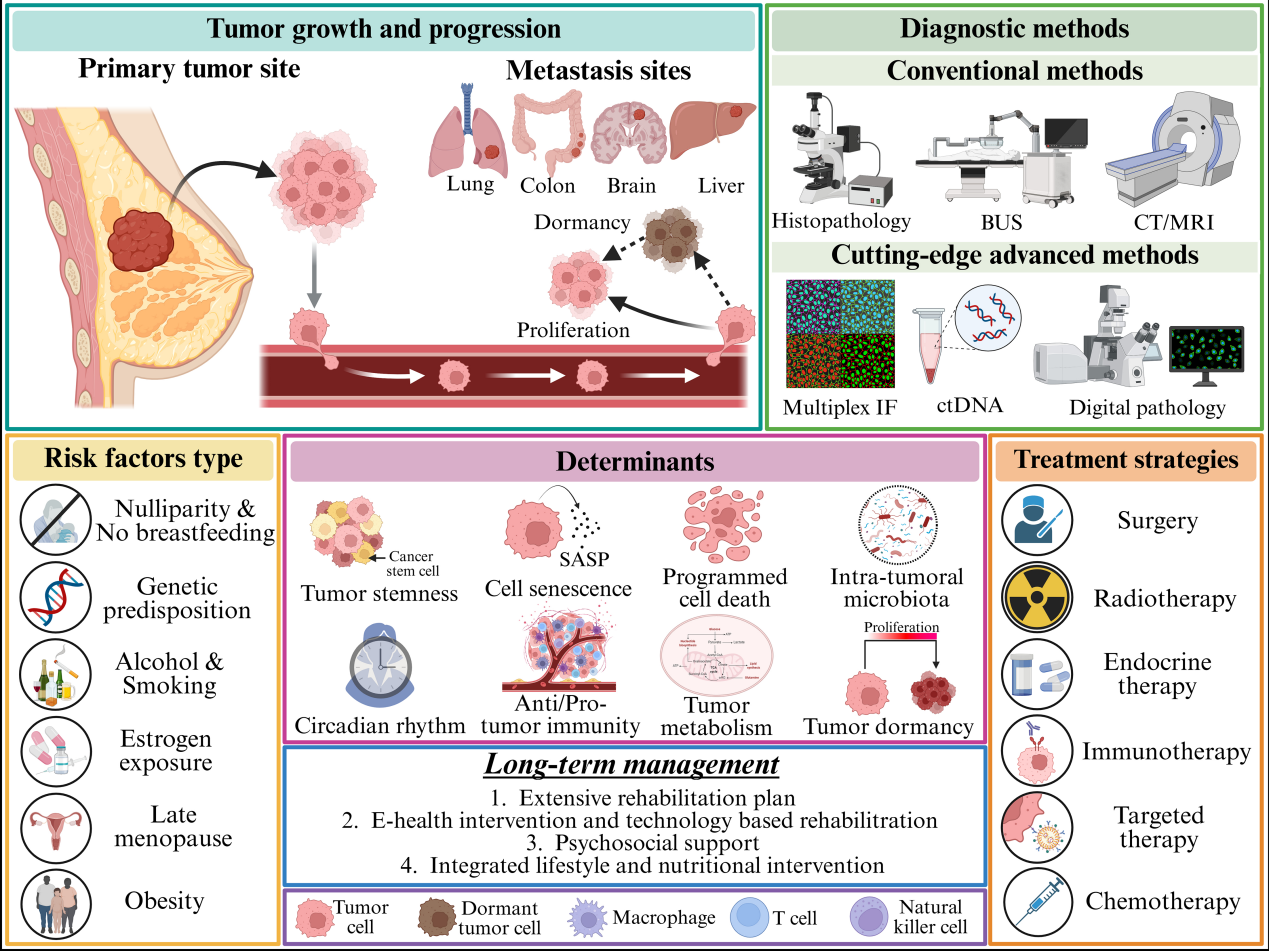
Breast cancer pathogenesis and treatment overview. Breast cancer is one of the most diagnosed cancers in women worldwide. In this figure we have shown its origin (primary breast tissue) and various sites where it can metastasize, such as the liver, brain, colon, lung, etc. Breast cancer occurs due to several factors, including genetic predisposition, nulliparity and no breastfeeding, hormonal factors (for example, estrogen exposure), lifestyle factors such as obesity, smoking, and alcohol consumption, and more. The tumor growth and progression are driven by several factors, such as tumor stemness, cell senescence, programmed cell death, microbiota present within and around tumors, circadian rhythm, immunological role, tumor metabolism, and tumor dormancy. Breast cancer diagnosis can be done using both conventional and advanced technologies such as histopathology, liquid biopsies (circulating tumor DNA, tumor-derived exosomes, etc.), imaging techniques (CT, MRI), immunofluorescence assay, digital pathology, etc. Breast cancer can be treated using several strategies, such as surgery, chemotherapy, radiotherapy, targeted therapy, immunotherapy, and endocrine therapy. Long-term management is very important, as it impacts patients’ overall health and survival outcome. BUS, B-scan ultrasonography; CT, computed tomography; MRI, magnetic resonance imaging; IF, immunofluorescence; ctDNA, circulating tumor DNA. BioRender.com was used to create the diagram.

This review provides a comprehensive picture and insight into the breast cancer research with emphasis on several risk factors, traditional and emerging trends in pathogenesis, diagnosis, subtype classification, and tailored treatment (**Figure 1**). In addition, the review discusses the contribution of computational approaches in fostering breast cancer research and the potential key challenges that require urgent attention.

## 2 Epidemiology and risk factors

Breast cancer is the most frequently diagnosed cancer globally and a leading cause of women’s death. In 2024 alone, it surpassed lung cancer as the most diagnosed cancer type worldwide, with over 2.3 million new cases every year (24). In India, it is the leading cancer diagnosed in the females, representing approximately 27% of all the cancers (25). This increase in number is because of aging populations, lifestyle changes, and enhanced monitoring and screening in both low- and high-income nations. Developed countries including United States of America, the United Kingdom, and Canada reported the maximum cases. However, the highest mortality was reported by the low- and middle-income countries including India, Brazil, because of a lack of proper treatment and advanced healthcare facilities for early diagnosis and screening (26, 27). Socioeconomic, racial, and ethnic distribution within countries further contributes to the variation in breast cancer pathogenesis. For instance, Yedjou et al. reported lower death rates in the United States in white women compared to African American and Hispanic women with higher mortality rates. In addition, African Americans have higher *BRCA1* frequency and are more prone to develop TNBC with poor clinical outcomes compared to women of European origin (28). Furthermore, Hispanic and Asian women develop breast cancer at earlier ages with different molecular subtype distributions and outcomes compared to white women (29).

The incidence rate of breast cancer also depends on age, as women in the USA and Canada on average develop it between 60 and 70 years, whereas women in countries of Asian origin, such as Japan and South Korea, develop it between 40 and 50 years (30). As per reports, the trend of developing breast cancer is becoming more popular in younger women (20–49 years) living in urbanized and industrial regions because of issues and challenges in early diagnosis due to limited pathology services and surgical capacity in areas including sub-Saharan Africa, South Asia, and parts of Latin America (31, 32). In India, as per GLOBOCAN 2022 report, incidence and mortality rates are higher among younger females (≤29 years) (1) and data obtained from National Cancer Registry Program by the national health agency Indian Council of Medical Research (ICMR) suggests that 5 per 100,000 individual develops breast cancer in rural region and 30 per 100,000 individual develops breast cancer in urban region (25).

Beyond race and ethnicity, reproductive characteristics play another major role in deciding the onset of breast cancer in countries such as Brazil, Egypt, and Nigeria, where women develop breast cancer with aggressive tumor biology and poor prognosis in their premenopausal state (33). This demographic change makes it essential to have tailored and customized public health policies that consider local burdens, genetic vulnerabilities, and the constraints of the healthcare system, ensuring that everyone receives fair access to diagnostics, risk reduction, and innovative treatments.

The development of breast cancer is associated with several factors, such as genetic predisposition, hormonal exposure, lifestyle changes, and environmental factors (**Figure 1**.).

### 2.1 Hormonal factors

Hormonal regulation is another important risk factor that plays a crucial role in breast cancer development, progression, and therapeutics (34). Female breasts respond strongly to hormones, and the interplay of such endogenous hormones, especially estrogen and progesterone, is a well-known major contributing factor in breast cancer carcinogenesis (35). A proper understanding of such hormone dynamics will allow us to have better insight into disease biology and clinical management, as the status of hormone receptors significantly influences treatment plans and outcomes (36, 37).

Estrogen, a steroid hormone, is predominantly synthesized in ovaries until menopause and in adipose tissue post-menopause (38). It has mitogenic effects on breast epithelial cells, and its long-term exposure via factors such as late menopause, menarche, nulliparity, or hormone replacement therapy (HRT) may put women at higher risk of developing breast cancer (39, 40). This phenomenon happens because of the ability of estrogen to stimulate cell division, which leads to higher chances of having DNA replication errors and mutations. In addition, estrogen metabolism can lead to reactive oxygen species (ROS) and genotoxic metabolites, which may cause DNA damage and tumor development (41, 42). Similar to estrogen, progesterone is another hormone essential for normal breast development and growth, yet it has a more complicated role in breast cancer (43). Progesterone can increase the estrogen property of cell division by activating the progesterone receptors, especially in tumors, which are hormone receptor-positive (44). Previous studies have shown that combined hormone replacement therapy (HRT) for ER-PR has been shown to accelerate the rate of breast cancer more significantly than estrogen-alone therapy (45). However, progesterone’s role is not limited to being oncogenic, as under certain conditions, it may exhibit differentiating or protective effects, depending on the isoform and cellular context of the receptor (46, 47).

Hormonal exposure is further modulated by several factors, such as reproductive factors. Women who have never given birth to an infant (nulliparity) or are at a very late stage are at higher risk compared to women who have given birth to multiple infants (multiparity) (48–51). Also, women with prolonged breastfeeding are at lower risk because of the suppression of ovulatory cycles and thus the reduction of levels of hormones (ER and PR) over time (52, 53). Another factor influencing hormone levels is the use of exogenous substances, such as oral contraceptives. They have been linked with a smaller but significant risk of developing breast cancer (54). However, the overall risk remains low and tends to diminish after some period of time post cessation of contraceptive use. Importantly, modern low-dose formulations of contraceptives have lower risk compared to the high dose that used to be given earlier (55).

Hormones further play a pivotal role in the molecular classification of breast cancer in different subtypes and the response to treatment based on their presence or absence (56–58). Mutations in the genes encoding estrogen hormone, *ESR1*, are associated with acquired resistance (intrinsic or extrinsic) to endocrine therapies (59). The hormone signaling is also influenced by a class of chemicals known as endocrine-disrupting chemicals (for e.g., bisphenol A (BPA) and phthalates), which bind to ER and alter the gene expression patterns (60, 61).

### 2.2 Genetic predisposition as risk factors

In the past 10–15 years, the genetic landscape of breast cancer has undergone substantial evolution, which highlights the properties of several genes that can drive hereditary susceptibility with moderate or high penetrance. Among many, *BRCA1* and *BRCA2* are the most extensively studied tumor suppressor genes (TSGs) linked with repairing double-strand DNA (dsDNA) breaks via homologous recombination. Germline mutations in *BRCA1/2* hamper the DNA repairing ability, progressing to genomic instability and malignant transformation. Mutation in *BRCA1* is associated with early onset and high-grade TNBC, whereas mutation in the *BRCA2* gene is more often responsible for estrogen receptor-positive (ER+) subtypes. Collectively, the risk of developing breast cancer in the *BRCA1* carriers in their lifetime is between 55% and 72%, whereas for *BRCA2* it falls in between 45% and 69%. Also, mutations in *BRCA1/2* make women more prone to develop ovarian cancer rather than breast cancer and men to develop pancreatic and prostate cancers, underscoring their systemic significance. Tumor cells with *BRCA1/2* mutations can be targeted using poly ADP ribose polymerase (PARP) inhibitors like olaparib and talazoparib (5, 62). In addition to BRCA genes, mutations in other high-penetrance genes such as *TP53* (associated with Li-Fraumeni syndrome) and *PTEN* (linked to Cowden syndrome) have been found to be associated with familial breast cancer, as these genes are heritable, keeping women at potential risk to develop cancer during their lifetime (5). Recently, other genes have emerged that, when mutated, can lead to a risk of developing breast cancer. These include *PALB2, ATM*, and *CHEK2* (5, 63).

The polygenic nature of breast cancer has been illuminated by the polygenic risk score computed based on multiple genome-wide association studies (GWAS), implicating over 180 single-nucleotide polymorphisms (64, 65). Although individual SNPs have little effect, their cumulative impact, especially in patients with a familial history of breast cancer, can significantly increase the patient’s risk category. NGS-powered multi-gene panel testing has now allowed researchers and clinicians to evaluate panels comprising *BRCA1, BRCA2, TP53, CDH1, ATM, PALB2, STK11*, and more, offering comprehensive genetic risk assessments (66).

Genetic risk factors and molecular subtypes together influence treatment outcomes. For instance, TNBC tumors with *BRCA1* mutations show better responses to chemotherapy initially; however, the relapse rate is higher (67, 68). In contrast, tumors with *ATM* or *CHEK2* mutations are usually estrogen receptor positive (ER+) and less aggressive (69, 70). As a result, genetic counseling is now an essential component of cancer treatment, directing treatment plans and family-centered measures like surveillance, prophylactic surgery, and cascade testing. Although only 5**–**10% of breast cancer is inherited, developments in molecular oncology and genetic screening technologies are allowing us to understand the role of genetic risk and develop personalized preventive and therapeutic plans for individual patients harboring pathogenic mutations (71).

### 2.3 Lifestyle factors

Lifestyle factors play an important and crucial role in shaping individual risk in developing breast cancer (72). In fact, these factors are associated with a significant proportion of breast cancer cases across the world, especially in high-income countries, where poor dietary habits (fast food, sugary food), alcohol intake, and sedentary lifestyles are highly prevalent. For detailed role and mechanism of various lifestyle factors in breast cancer, kindly refer to the previous studies (73–85). A deeper understanding of these above-mentioned risk factors will help in personal risk assessment as well as lay a strong foundation for developing public health strategies aimed at prevention.

### 2.4 Environmental factors

Other than genetic, hormonal, and lifestyle risk factors, environmental exposures have emerged as another significant contributor to breast cancer risk (35). The common environmental factors include ionizing radiation; widespread use of EDCs such as BPA, phthalates, and pesticides such as DDT (86); air pollution including fine particulate matter (PM2.5), polycyclic aromatic hydrocarbons (PAHs), and nitrogen dioxide (NO2); chemicals such as per- and polyfluoroalkyl substances (PFAS) (87); change in the natural circadian rhythm (88); occupational hazards such as benzene and formaldehyde; and persistent organic pollutants (POPs) such as dioxins and organochlorines (89). These exposures act via a mechanism involving hormonal dysregulation, oxidative stress buildup, DNA damage, and epigenetic alterations.

### 2.5 Reproductive, nutritional, ethnic and sociocultural factors

Dietary patterns play a critical role in modulating the risk of breast cancer occurrence. As per previous studies, it has been noted that an increased intake of processed foods, red meat, and saturated fats contributes to the risk of breast cancer occurrence (90), while fruits and vegetables with a high constituent of bioactive compounds have been noted to have a protective role (91).

Extensive studies have demonstrated that breastfeeding reduces lifetime risk for developing breast cancer through hormonal control, delayed ovulation, and mammary epithelial cell differentiation, with the longer the duration, the more protective effect (92).

The variation in incidence rates of breast cancer between ethnic groups is already well established, including variations between patient subtypes, age of onset, and survival rates for people of both European, African, and Asian descent (93, 94). Moreover, recent studies are indicating that biological pathways that are melanin-related and the metabolism of vitamin D, which are also affected by complexion, can play a role in the disease, especially for darker-complexioned women (95).

As far as the use of a garment, i.e., a brassiere, is concerned, a number of studies have found that there was no association between the use of a bra, the duration, and the cup size and the development of breast cancer, indicating that there was no scientific evidence that supported the notion that there was a causal relationship (96).

## 3 Molecular classification of breast cancer

Pathologically, breast cancer is classified into two major categories: (i) breast invasive carcinoma, which accounts for 70-75% of the cases, and (ii) lobular carcinoma, accounting for 12-15% of the cases. Moreover, for accurate prognostic evaluation and clinical decision-making, breast cancer is grouped into four groups. This classification is based on immunohistochemistry (IHC) staining results and expression of hormone receptors ER, PR, and HER2, along with key proliferation markers such as Ki-67 (35), and allows designing and prescribing personalized treatment, as each subtype exhibits distinct biological properties, therapy response, and prognosis. Below we have discussed the intrinsic molecular subtype in detail.

### 3.1 Luminal A subtype

Luminal A is the most prevalent form of breast cancer subtype, accounting for nearly 40–50% of the cases. It is characterized by the ER and PR presence, HER2 absence, and low proliferation index quantified by low level of Ki67 (7). The key hallmark properties of the Luminal A subtype include favorable prognosis, slow tumor growth, lesser likelihood of lymph node involvement, and high response to endocrine therapy such as tamoxifen or aromatase inhibitors (97). The gene expression profile of the Luminal A subtype is linked with processes such as luminal epithelial cell differentiation, estrogen-related pathways, etc. Though the subtype responds well to endocrine therapy, exceptions exist, particularly in cases where the expression of Ki67 is elevated, suggesting a shift towards a more aggressive phenotype. In addition, in the genomics context, Luminal A subtypes possess fewer mutations and chromosomal aberrations (98).

### 3.2 Luminal B subtype

The luminal B subtype is more aggressive than luminal A, accounting for nearly 20–30% of the breast cancer cases. The hallmark features of the subtype include higher expression of Ki67 (hence higher proliferation rate), lower expression of PR, and in some cases, positive status of HER2 and ER, a more aggressive phenotype with higher chances of early relapse, poor prognosis, lower survival rate, lymph node involvement, and metastasis (99). Tumors in this subtype are usually resistant to endocrine therapy alone and only benefit when given additional treatments like chemotherapy or targeted therapy (97). Additionally, they show elevated pathways related to cell division and growth mediated by cyclins and cell cycle regulators. From a genomics perspective, luminal B subtype tumors show higher genomic instability and mutations (98).

### 3.3 HER2 subtype

This class of tumors is characterized by increased HER2 expression, while ER/PR expression is absent. They form about 15-20% of all breast cancer cases. The characteristics are aggressive nature, involvement of lymph nodes, early recurrence, and a poor prognosis unless anti-HER2 therapy, like trastuzumab, pertuzumab, and ado-trastuzumab emtansine, is provided. These drugs have helped to significantly improve the outcome for HER2 patients (100, 101). Key hallmarks of tumors classified as the HER2 subtype include higher expression of hormone receptors, higher proliferation rates, and extensive genomic instability (102). HER2 overexpression drives cancer cell proliferation by activating several downstream signaling pathways, including MAPK, PI3K/AKT, and more. Tumors in this group tend to be of high grade and get metastasized in the early time course of the disease.

### 3.4 Triple negative breast cancer

TNBC is referred as the most aggressive form of breast cancer and is characterized by the absence of all three hormone receptors, i.e., ER, PR, and HER2. This cancer is common in women under 40 years of age and constitutes nearly 10–15% of breast cancer cases. TNBC is associated with higher histologic and nuclear grades and a poorer prognosis compared to other subtypes (103). TNBC is further divided into four subtypes, i.e., BL1, BL2, LAR, and M (104). TNBC is a heterogenous form of breast cancer and becomes more aggressive when they exhibit basal-like features, which may confer some advantage from chemotherapy, particularly anthracycline-based regimens. Understanding TNBC molecular and histopathological features will allow us to design more effective therapeutic strategies and predict patient outcomes.

**Figure 2:**
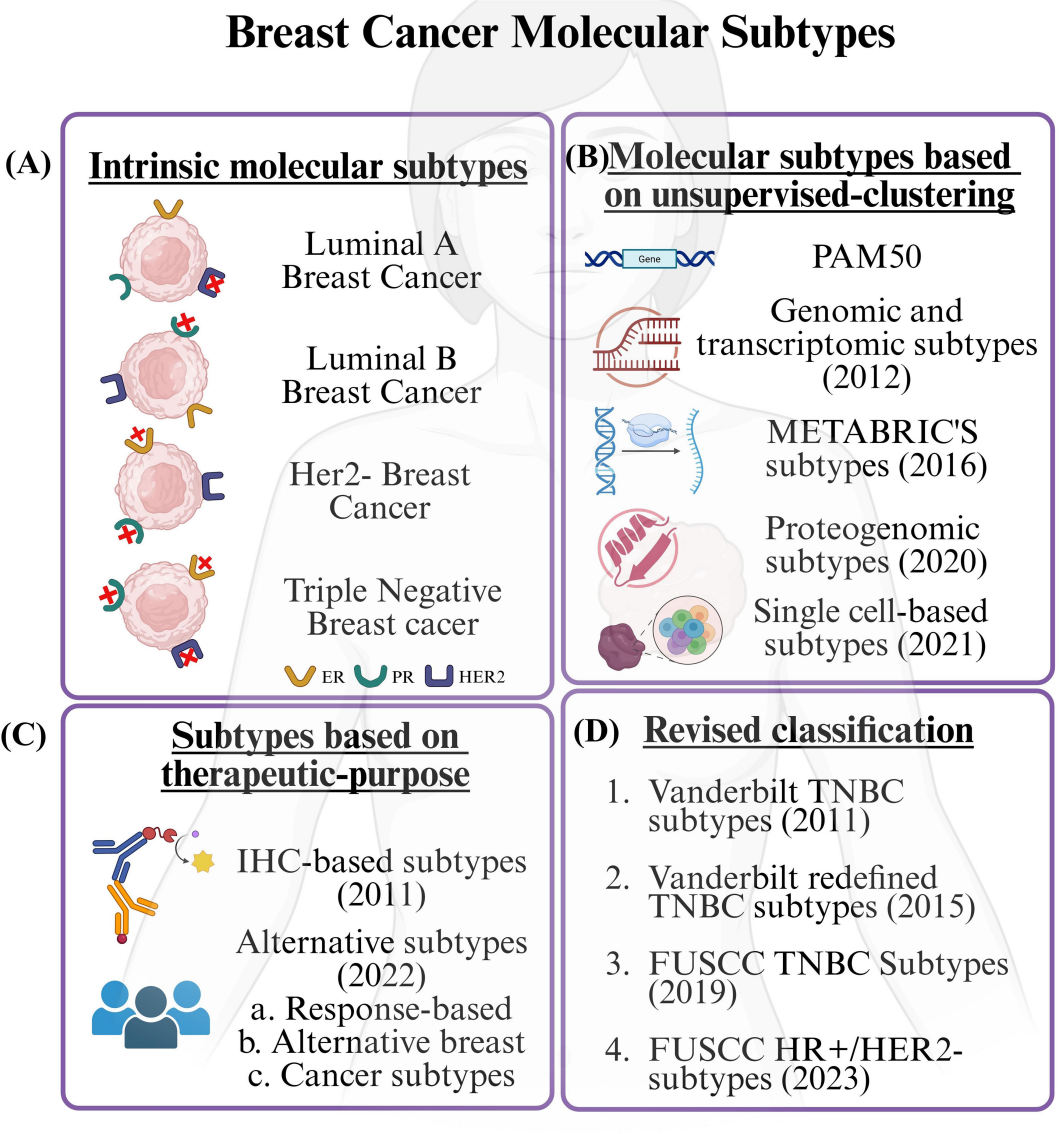
Breast cancer classification. Breast cancer has been classified based on several factors. In this diagram, we have shown different classifications proposed over the years based on several factors. **(A)** In the first section, we present the intrinsic molecular subtypes, which are mainly defined by gene expression profiling (e.g., PAM50), but they are commonly written and reported in clinical practice using immunohistochemical (IHC) markers, i.e., Estrogen Receptor (ER), Progesterone Receptor (PR), and Human Epidermal Growth Factor Receptor (HER2). Based on the presence or absence of these markers, this subtype is classified as luminal (A/B), HER2, or basal or triple-negative breast cancer (TNBC). In section **(B)** we reported subtypes of breast cancer that have been proposed based on unsupervised machine learning techniques. These include intrinsic subtypes, the widely accepted PAM50 classification, subtypes derived from genomic and transcriptomic data, METABRIC’s subtype, proteogenomic subtypes (which are based on protein and post-translational modifications), and advanced subtypes such as those identified through single-cell analysis. **(C)** Breast cancer subtypes have also been proposed as per patients’ responses to various treatments. Two such common subtypes include the IHC-based subtype and alternative subtypes. **(D)** Lastly, we have subtypes that have been reclassified based on parameters such as gene expression, proliferation signatures, etc. Common ones include the classification proposed by Vanderbilt for classifying TNBC subtypes, FUSCC TNBC subtypes, and FUSCC HR+/HER2- subtypes. IHC, Immunohistochemistry; TNBC, Triple Negative Breast Cancer; FUSCC, Fudan University Shanghai Cancer Center; HR, Hormone Receptors; HER2, Human Epidermal Growth Factor Receptor 2. BioRender.com was used to create the diagram.

Beyond gene expression and receptor status, other factors such as proteomics, post-translational modifications, epigenomics, and cell-type-specific expression have been used to stratify breast cancer into subtypes. In addition, responses to therapy have been used to classify breast cancer into different subtypes (**Figure 2**).

## 4 Pathogenesis and tumor microenvironment

Breast cancer is an ecosystem that contains both malignant cells and stromal cells and are characterized by a substantial change in the surrounding stroma, or TME, essential for cancer growth and progression. Chronic inflammation, ECM remodeling, immune cell infiltration, and hypoxia-driven angiogenesis are some of the key processes associated with breast cancer pathogenesis. All of these factors are interrelated, and their roles are discussed in brief below.

### 4.1 Inflammation and immune cells

TME of breast cancer is associated with chronic and tumor-promoting inflammation. Infiltrating immune cells either exhibit anticancer responses or create an immunosuppressive environment (105). TILs, particularly cytotoxic CD8+ and Th1-type CD4+ T cells, have shown improved prognosis and therapeutic response, especially in TNBC and HER2-positive breast malignancies. In contrast, M2 polarized tumor-associated macrophages (TAMs), are associated with worse outcomes as they cause angiogenesis, ECM degradation, and metastasis (106). Furthermore, myeloid-derived suppressor cells (MDSCs), regulatory T cells (Tregs), and dysfunctional dendritic cells further impede CTL activity. An “immune-hot” TME with multiple CD8+ T cells prevents tumor growth; in contrast, an “immune-cold” TME with MDSCs, TAMs, and Tregs allows tumor growth and progression. This immunosuppressive environment is maintained by several cytokines such as IL-6 and TNF-α, which activate NF-κB/STAT3 signaling, and chemokines such as CCL2 and CXCL12, which bring in monocytes (107, 108). These monocytes further get differentiated into TAMs, which in turn secrete factors such as EGF, VEGF, and TGF-β, driving tumor invasion, spread, and new blood formation (109). The above process creates a self-perpetuating inflammatory loop and sustains tumorigenesis.

### 4.2 Extracellular matrix remodeling

ECM plays an important role in breast cancer pathogenesis and is remodeled by both cancer and stromal cells, particularly by cancer-associated fibroblasts (CAFs). Tumors derived growth factors like TGF-β and platelet-derived growth factor (PDGF) activates the fibroblasts present within the CAFs, which in turn release abundant ECM proteins, leading to desmoplasia with abundant collagen I/III, fibronectin, and other fibrillar matrices (110). Crosslinking collagen creates a stiffened and aligned matrix, creating migration tracks for cancer cells. This crosslinking of collagen and elastin is carried out by an enzyme known as lysyl oxidase (LOX), which is induced by hypoxia (via HIF-1α) and oncogenic signaling, leading to invasion and therapy resistance. Inhibiting the LOX enzyme allows loosening of matrix stiffness, fibronectin disruption, and improved drug penetration (111). In contrast, members of another class of enzymes, matrix metalloproteinases (MMPs), such as MMP-1, -2, -9, -11, and -14, decompose basement membranes and interstitial matrix, releasing growth factors such as VEGF and TGF-β. These factors are associated with the angiogenesis promotion and EMT. Expression of MMPs present in abundance in breast cancer stroma, such as MMP-11 and MMP-14, is linked with a poor prognosis (112). Additionally, CAFs have been found to secrete glycoproteins such as tenascin-C, periostin, and proteoglycans such as versican, which regulate cell adhesion and proliferation (110). Integrin α5β1–FAK/Src signaling is activated by alteration in the ECM composition, which in turn enhances the motility, while lymphocyte infiltration is restricted by the dense collagen network, adding another layer of immunosuppression (113). Overall, dynamic ECM remodeling results in a stroma that is mechanically and biochemically aberrant and promotes tumor progression.

### 4.3 Hypoxia and angiogenesis

Uncontrolled division of breast cancer cells often outgrows their blood supply, creating hypoxic conditions (low oxygen level). This hypoxic environment in turn stabilizes HIF-1α in both cancer and stromal cells (114). HIF-1α regulates expression of numerous factors linked with angiogenic processes (particularly VEGF), glycolytic enzymes, and pro-metastatic genes. In breast cancer, particularly in TNBC, HIF-1α binds to the VEGF promoter directly and activates VEGF transcription (115). The binding leads to stimulation of endothelial cells and triggers pathways such as MAPK/ERK, PI3K/Akt, and PLCγ signaling to drive proliferation, migration, and tube formation. The resulting vasculature is pain-giving, dilated, and leaky, with poor pericyte coverage, which in turn worsens hypoxia and stabilizes HIF-1α signaling in a vicious cycle (116). Beyond angiogenesis, HIF-1α advances invasion via EMT induction and upregulates matrix remodelers such as LOX and MMPs, associating with metastasis and poor prognosis in breast cancer. VEGF also regulates and shapes immunosuppressive TME by inhibiting dendritic cell maturation, preventing antigen presentation, and by polarizing macrophages toward an M2 phenotype. In addition, high levels of VEGF and HIF-1α are linked with resistance to therapy and metastatic progression in clinical settings. In conclusion, the hypoxia–HIF‐1α–VEGF pathway regulates inefficient vasculature, which sustains tumor cell metabolism, facilitates dissemination, and reinforces immunosuppression (117, 118).

### 4.4 Cancer associated fibroblasts

Cancer-associated fibroblasts (CAFs) are among the most prevalent stromal cells in the breast cancer TME and have a pivotal role in the initiation, development, and resistance of cancer to therapies (119). CAFs upon activation secretes growth factors, cytokines, and ECM molecules including TGF-β, matrix metalloproteinases (MMPs), hepatocyte growth factor (HGF), and fibroblast growth factors (FGFs), which are implicated in tumor development, invasion, and metastasis (120). CAFs-induced ECM remodeling results in increased tissue stiffness and the activation of mechanotransduction signaling pathways, such as integrin-FAK and YAP/TAZ signaling, thereby conferring increased malignancy to tumors (121). Moreover, CAFs modulate the infiltration and polarization of immune cells, thereby creating an immunosuppressive microenvironment that suppresses antitumor immune responses (120). Recent studies also indicate that CAFs modulate tumor metabolic support by the transfer of nutrients and metabolic symbiosis, thereby contributing to the maintenance of cancer cell growth (121).

### 4.5 Metabolic reprogramming and adipocyte crosstalk

Metabolic reprogramming is one of the hallmarks of the breast cancer progression and is strongly influenced by the complex interactions among tumor cells and nearby adipocytes present within the TME (122, 123). Cancer-associated adipocytes exhibit phenotypic changes including secretion of the lipids, inflammatory cytokines, and an imbalance in adipokine signaling pathways, such as the upregulation of leptin and downregulation of adiponectin, which promote tumor proliferation and invasion (123). The free fatty acids, which are transported from adipocytes to cancer cells, are an important source of energy for the rapid growth and metastasis of cancer (122). At the same time, metabolic shift towards aerobic glycolysis, altered lipid metabolism, and glutamine dependency due to oncogenic signaling and hypoxic stress, further promotes tumor survival and therapy resistance (123). This metabolic relationship creates nutrient-rich microenvironment and supports the breast cancer growth, especially in cases associated with obesity.

### 4.6 Extracellular vesicles and exosome-mediated signaling

Extracellular vesicles (EVs), especially exosomes, plays a crucial role in mediating intercellular communications with the TME of breast cancer (124). Exosomes originated from tumor cells carries several cargoes such as oncogenic proteins, microRNAs, and metabolites that reprogram stromal cells to support angiogenesis, suppression of immune system and tumor growth (125). These small vesicles plays a major role in the formation of pre-metastatic niche by modifying distant organs (brain, liver, lung, etc.) and enhancing metastatic organotropism (124). In case of breast cancer, several exosomal microRNAs (exo-miRs) such as miR-21, miR-105, miR-20b have been found to enhance EMT, chemoresistance, and immune evasion (126). Likewise, exosomes derived from stromal cells reinforce tumor progression by transporting growth signals and metabolic substrates, underscoring the bidirectional nature of EV-mediated communication within the TME (125).

### 4.7 Vascular niche and endothelial cell signaling

In addition to angiogenesis, endothelial cells play an important role in the regulation of breast cancer progression by forming a vascular niche which promotes the survival, stemness, and metastatic spread of tumor cells (127). Endothelial cells associated with tumors secrete growth factors such as vascular endothelial growth factor (VEGF), Notch ligands, and nitric oxide, which enables the cancer cells proliferation and self-renewal (127). The abnormal architecture of the tumor vasculature results in hypoxia, immune exclusion, and poor drug delivery, thus promoting therapeutic resistance (128). Additionally, the enhanced vascular permeability promotes the intravasation of tumor cells and metastasis. Recent studies indicates that vascular normalization approaches may improve therapeutic efficacy by enhancing perfusion, immune infiltration, and drug delivery (129, 130).

## 5 Mechanisms driving breast cancer

**Figure 3:**
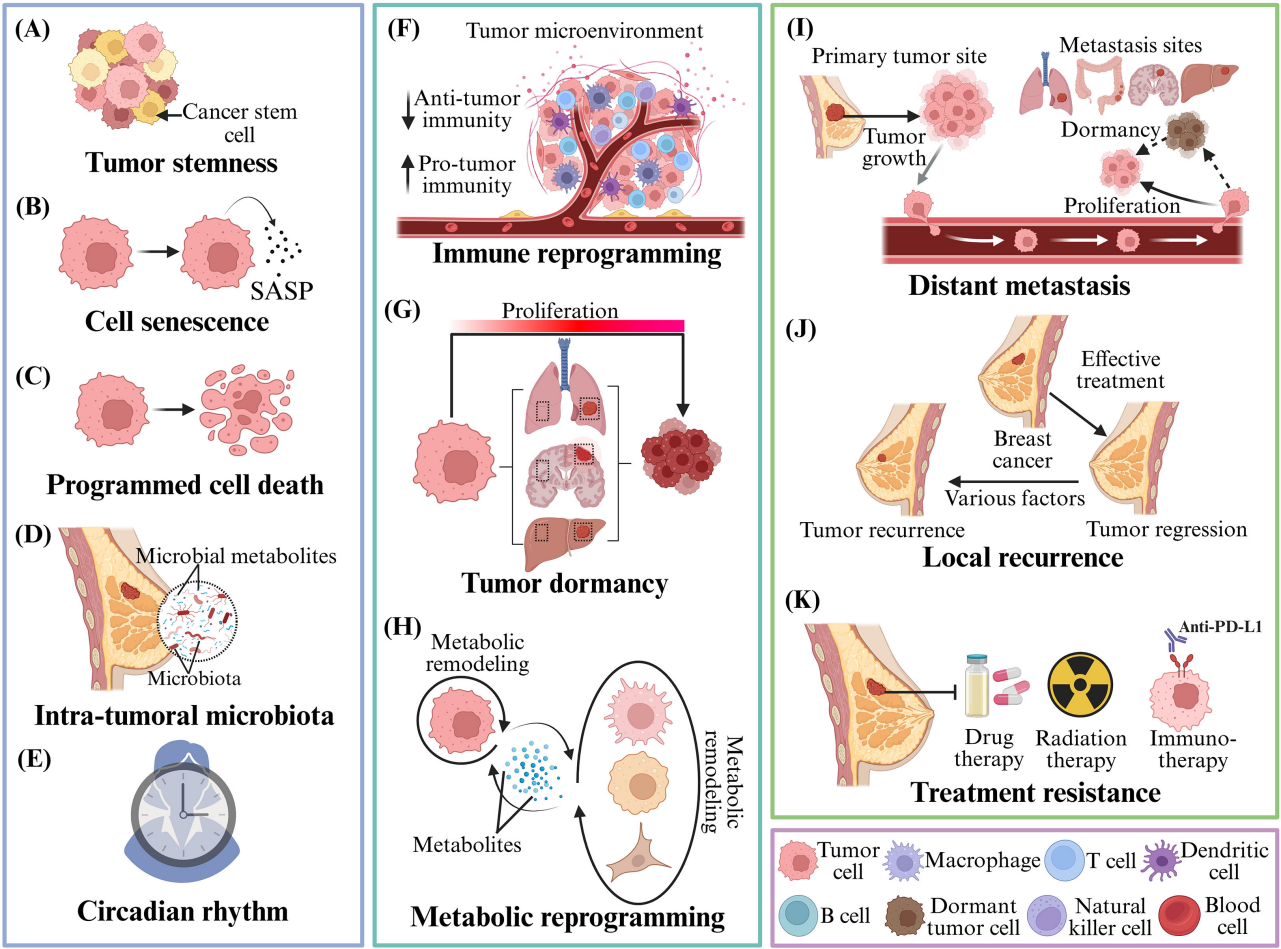
Breast cancer progression regulating factors. Numerous factors contribute to breast cancer progression, including **(A)** tumor stemness, such as the role of cancer stem cells; **(B)** cell senescence, such as the role of SASP; **(C)** programmed cell death; **(D)** microbiota and microbial metabolites; **(E)** circadian rhythm; and **(F)** the role of immune programming. **(G)** Tumor dormancy; **(H)** Metabolic reprogramming. These factors are responsible for **(I)** local tumor recurrence, **(J)** metastasis to distant organs, and **(K)** resistance to treatment. SASP, Senescence Associated Secretory Phenotype. BioRender.com was used to create the diagram.

For any type of cancer, including breast, the progression of the tumor is characterized by local recurrence, metastasis, and the development of therapy resistance, all of which pose a challenge that needs urgent attention. Advancements in experimental protocols and sequencing technologies over the time have led to significant achievements in understanding the underlying mechanisms driving breast cancer. Below, we highlight some of the key factors contributing to the progression of breast cancer (**Figure 3**) and detailed list is provided in **Table 1**.

**Table 1:** Factors associated with breast cancer progression.

Mechanism	Biomarker/gene	Role	Ref.
Tumor stemness	CT83 (KK-LC-1)	Inhibits FAT1-Hippo pathway; promotes YAP1 nuclear translation subsequently ALDH1A1 transcription	(131, 132)
	EMSY	Binds to KDM5B Jmjc domain and reshaping methionine metabolism in CSCs	(133)
	DKK1	Increase the expression of SLC7A11 and inhibits tumor metastasis by lipid peroxidation and ferroptosis	(134)
	PCAT6	Enhances nuclear mRNA export to BCSCs	(135)
	THOC	Acts as a nuclear mRNA export regulators and increases pluripotency factor expression, such as NANOG and SOX2	(136)
Cellular senescence	TNFRSF11A (RANK)	RANK overactivation in mammary epithelium cause delays in tumor onset	(137)
	CD274 (PD-L1), CD80	SASP-associated immune evasion and fosters immune escape	(138, 139)
	TSPAN8, IL-6, IL-8	Senescence like TSPAN8 and myCAF potentiates tumor stemness through SASP-associated factors IL-6 and IL-8	(140)
Cell death	Ferroptosis	Characterized by iron-dependent accumulation of lipid peroxides; Inhibitors of glutathione peroxidase 4 (GPX4) regulates the process in TNBC.	(141, 142)
	Cuproptosis	Copper-driven form of PCD that results in mitochondrial proteotoxic stress due to excess intracellular copper, mainly mediated by FDX1	(143, 144)
	Disulfidoptosis	Occurs due to SLC7A11 overexpression leading to excessive cystine uptake and depletion of NADPH, resulting in aberrant actin disulfide bonding, cytoskeletal disruption, and cell death	(145)
Microbiome within tumors	Clostridiales	Bacterial genus observed within TNBC cells along with high levels of metabolite, trimethylamine N-oxide (TMAO), associated with pyroptosis of tumor cells via PERK-dependent ER stress.	(146)
	Fusobacterium nucleatum	Translocates to breast cancer cells via metastasis. It attenuates T-cell infiltration to the tumor, thus aiding tumor development.	(147)
Circadian rhythm	CXCL12-CXCR4 axis	Contributes to the formation of an immunosuppressive microenvironment, CXCR2 inhibitor counteract dissemination and metastasis	(148)
Metabolic reprogramming	SLC5A6 (SMVT)	MYC regulates SLC5A6, promoting intracellular transport of Vitamin b5 and its conversion to coenzyme A therefore enhance TCA and fatty acid biosynthesis	(149)
	DNM1L (DRP1)	Promotes mitochondrial fission leading to fatty acid oxidation metabolism	(150)
	SLC7A11 (xCT)	Glutamine utilization and oxidative stress response	(151)
Immune reprogramming	PIK3CB (PI3Kb)	Restricts CD4^+^/CD8^+^ T cells infiltration via VBMX/STAT3 signaling pathway	(152)
	DDR1	ECM collagen fiber alignment barrier	(153)
	NAT8L	High N-acetylaspartate impaired immunological synapse, induces NK and CD8^+^ T cells dysfunction	(154)
	FGF21	Alters cholesterol metabolism in CD8^+^ T cells and therefore drive CD8^+^ T cell exhaustion	(155)
	COPI	Regulates macrophage chemokine secretion and promotes M2 macrophage infiltration, inhibition of enhances anti-PD1 response	(156)
	ICOSL^+^ B cell	Promote T-cell dependent antitumor immunity therefore enhances the efficacy of chemotherapy	(157)
	HSPA4	Promote the expression of CXCR4 and secretion of SDF1a, activate NF-kB pathway resulting expression of HIF1a and COX2	(158)
Tumor dormancy and reactivation	COL3A1	Cause ECM switch controlling dormancy *vs* proliferation; disruption activates DDR1-STAT1 to drive tumor cell proliferation	(159)
	CXCL12-CXCR4	aHSC CXCL12 induce CXCR4 on NK cells, induce NK cell quiescence, loss of IFN-g surveillance reactivates DTCs	(160)
	PDGFC	Promote fibroblast activation in senescent and fibrotic lung	(161)
	NR2F1-AS1	Facilitates local diffusion while inhibiting lung metastasis activation	(162)
	DAG1	Astrocyte-derived laminin 211 binds to DAG1 resulting DTCs quiescence.	(163)
	NFE2L2	Redox and nucleotide metabolism reprogramming	(164)
	ZFP281	FGF2 and TWIST1 regulate ZFP281; induces cadherin-11; drives EMT and dissemination	(165)

In this table, we have provided the various factors associated with breast cancer progression, different biomarkers under each category, and their potential role.

- Tumor stemness: Tumor stemness represents the capacity for cancer cells to display self-renewal, differentiation, and tumor-initiation potential, identical to regular stem cells. Tumor stemness is exhibited by a subset of cancer cells, known as cancer stem cells (CSCs). Numerous reports substantiate that CSCs play a crucial role in driving tumor initiation, treatment resistance, recurrence, and metastasis (**Figure 3A**). CSCs are commonly characterized by using different markers such as CD133, CD44, EPCAM and ALDH1. The first report on solid tumors was made only in breast cancer, where Kita-Kyushu lung cancer antigen-1 (KK-LC-1) was characterized as a novel biomarker for TNBC CSCs. KK-LC-1 binds to FAT1 and inhibits Hippo signaling (131). Likewise, EMSY promote self-renewal through metabolic reprogramming linked to H3K4 methylation (133). In addition, suppression of FBXL2, a stemness suppressor further increases chemoresistance, and FBXL2 re-activation restores drug sensitivity (166). DKK1 which is derived from CSC paradoxically suppresses population of stem cells yet leads to metastatic survival through SLC7A11-mediated ferroptosis resistance (134). Furthermore, PCAT6 and THOC proteins, acting as a nuclear mRNA export regulators, increases pluripotency factor expression, such as NANOG and SOX2 (135, 136). Apart from intrinsic signaling, the TME shapes CSCs behavior and mechanical cures from ECM further modulates CSC fate, with poor stiffness enhancing stemness and high stress leading to quiescence via distinct integrin-dependent pathways.
- Cellular senescence: It is a form of self-defense mechanism where there is a pause in cell division, but there isn’t permanent death. Senescence is triggered by internal as well as external stimulation, playing a pivotal role in organismal growth and post-injury repair. Primary hallmarks of cellular senescence include cell-cycle arrest, resistance to cell death, and senescence-associated secretory phenotype (SASP) (**Figure 3B**). The process exhibits a dual role in tumorigenesis by promoting as well as suppressing tumors (167). Overactivation of the RANK signaling pathway in Neu and MMTV-PyMT oncogenic mouse models delays breast cancer onset and promotes subsequent metastatic invasion (137). Certain tumor cells during breast cancer chemotherapy show upregulation of SASP genes, followed by an augmented expression of PD-L1 and CD80, immunosuppressive molecules, within cancer cells. This phenomenon leads to immune evasion, allowing tumor cells to survive during chemotherapy (168). In addition, multiple other factors directly or indirectly regulate cellular senescence, including the role of CAFs within TME, tetraspanin-8 (TSPAN8)+ myofibroblastic CAF (myCAF), and senescent neutrophils (140).
- Different kinds of programmed cell death: A process known as “cell death” in a given organism carries out the development and maintenance of homeostasis. It can be broadly categorized into two groups:(a) accidental cell death and (b) regulated cell death (RCD), popularly known as programmed cell death. Programmed cell death (PCD) is further comprised of several forms, including apoptosis, pyroptosis, ferroptosis, and entotic cell death. These forms act via distinct mechanisms and are associated with cancer progression and metastasis (169–171) (**Figure 3C**). Ferroptosis, a natural form of RCD, is characterized by iron-dependent accumulation of lipid peroxides. The process not only impacts cancer cells but also regulates antitumor immune response. Among the four TNBC subtypes, LAR (luminal androgen receptor) exhibits the highest ferroptosis activity. The process is regulated by the inhibitors of glutathione peroxidase 4 (GPX4), one of the key factors, which block the cancer cell proliferation and enhance antitumor immunity. Combining GPX4 with immunotherapy can further prevent the tumor progression (172, 173). Cuproptosis is a copper-driven form of PCD that results in mitochondrial proteotoxic stress due to excess intracellular copper, mainly mediated by FDX1, and has been demonstrated to possess tumor-suppressing properties in cancer models (143, 144). Likewise, disulfidoptosis occurs due to SLC7A11 overexpression leading to excessive cystine uptake and depletion of NADPH, resulting in aberrant actin disulfide bonding, cytoskeletal disruption, and cell death, especially during glucose deprivation (145). Both, cuproptosis and disulfidoptosis, utilize redox imbalance, and agents targeting intracellular redox homeostasis may activate multiple pathways of cell death to better inhibit tumor progression (141).
- Microbiome within tumors: Contrary to normal tissues which are usually sterile, the presence of bacterial and fungal species has been observed within tumor and immune cells, associated with remodeling TME and actively promoting tumorigenesis. Bacteria in breast cancer, particularly from the genus Clostridiales have been observed within TNBC cells along with high levels of an associated metabolite known as trimethylamine N-oxide (TMAO). TMAO is associated with pyroptosis of tumor cells via PERK-dependent ER stress, improving antitumor immune response via CD8^+^ T cells. These tumor cells, when they metastasize, carry bacteria to distant organs, promoting cytoskeletal reorganization and resistance to shear stress in the bloodstream, aiding metastasis survival (146). Likewise, another bacterium, *Fusobacterium nucleatum*, closely linked to colon cancer cells, is often translocated to breast cancer cells via metastasis. *F. nucleatum* have been demonstrated to attenuate T-cell infiltration to the tumor, thus aiding tumor development (147) (**Figure 3D**).
- Circadian rhythm: Circadian rhythm (CR), also known as biological clock, is a 24-hour cycle that coordinates immune function and metabolism in response to various signaling pathways within the body and the external factors such as temperature, light, etc. The CR process regulates the immune and physiological function, and any disruption in it leads to severe diseases, including cardiovascular, neurodegenerative, cancer, etc. Disruptions in this process have been reported in cases of breast cancer (**Figure 3E**). It not only increases the tumorigenesis potential but also creates an immunosuppressive environment for tumor metastasis by impacting chemokine/chemokine receptor signaling (CXCL12-CXCR4 being the primary one). Pharmacological inhibition of CXCR2 can partially reverse metastasis driven by chronic circadian disruption. Furthermore, disruption in the CR process leads to the production of higher CTCs, particularly during sleep time, which can further promote the tumor progression as these CTCs display elevated expression of circadian hormone receptors, and hormones such as melatonin modulate their release (174, 175). Single-cell transcriptomic profiling of CTCs collected during resting phases reveals elevated mitotic activity, providing a mechanistic basis for time-dependent metastatic potential and underscoring the importance of chronobiology in cancer diagnosis and therapy (176).
- Immune reprogramming: A proper functioning immune system is crucial for tumor cell death and elimination. However, tumor cells can remodel TME by several means, including increased immunosuppressive population, reduced infiltration of tumor-inhibitory immune cells, reduced presentation of tumor antigens on MHC molecules, and more, allowing them to evade the immune system (**Figure 3F**). However, the precise mechanisms by which tumor cells evade the immune system remain incompletely understood. In case of breast cancer lacking *PTEN*, *PI3KB* expression in cancerous cells alters the CD4+ and CD8+ T-cell infiltration via the BMX/STAT3 signaling pathway, facilitating the “immune desert” formation within tumors and promoting tumor immune evasiveness (152). Additional restriction to immune access is conferred by physical barriers of structural ECM remodeling, whereby tumor-derived discoidin domain receptor 1 fragments realign collagen fibers, forming physical barriers that impede immune cell penetration (177). Beyond immune exclusion, immune function is actively suppressed by tumors: elevated N- acetylaspartate (NAA) production disrupts the immunological synapse in CD8^+^ T and NK cells (178), while FGF21 derived from cancer cells rewires cholesterol metabolism toward inducing T-cell exhaustion (155). E3 ligase Cop1, an essential regulator of macrophage chemokine secretion was identified within breast cancer cells by researchers using large scale CRISPR screen experiments. This Cop1 is responsible for driving TAM recruitment, M2 polarization, and promoting resistance to immunotherapy and its inhibition leads to enhanced antitumor responses to PD-1 blockade (156). Immune reprogramming is also systemic, as shown by reduced cytotoxic immunity and increased immunosuppression in pre-metastatic lungs and distant metastatic sites (179–181). Notably, B cells have been shown to also harbor dual roles: chemotherapy-induced ICOSL^+^ B cells enhance T-cell–mediated antitumor immunity (157), while pathological antibodies activate NF-κB–HIF1α–COX2 signaling in tumor cells to form pro-metastatic lymph node niches (158).
- Disruption in tumor dormancy and reactivation: Disruption in tumor dormancy and reactivation of long-persisting disseminated tumor cells (DTCs) in distant organs post-primary surgery constitutes a crucial role in tumor relapse and confers a significant challenge in anti-cancerous therapy (**Figure 3G**). Tumor relapse, in the case of breast cancer, usually happens between 5- and 20-years post-treatment, especially in tumors that are ER+ (182). Previous research has revealed specific mechanisms linked with tumor dormancy and reactivation, while newer findings have further enhanced our understanding. For instance, in breast cancer, collagen type III is responsible for ECM switch, and any disruption will lead to tumor cell division via DDR1-mediated STAT1 signaling (159). Likewise, crucial role of immune cells has been seen in tumor dormancy, as NK cells which have been found to maintain DTC dormancy in the liver through IFN-γ production. Conversely, activated hepatic stellate cells have been reported to suppress NK cell function through the CXCL12/CXCR4 signaling cascade (161). At the same time in the lung, senescence or fibrosis induced PDGF activates fibroblasts, reactivating DTCs (162). Dormancy is further stabilized by intrinsic regulators such as lncRNA NR2F1-AS1, which regulates stemness to prevent metastatic activation, and laminin-211 from the astrocytes in the brain, which maintains quiescence through dystroglycan signaling (163). Reactivation is also governed by metabolic reprogramming where NRF2-mediated redox and nucleotide synthesis drive tumor reemergence (164). Finally, pluripotency factor such as ZFP281 has been observed to orchestrate early metastatic dormancy by enabling dissemination through EMT and concurrently imposes long-term quiescence in distant sites, describing non-traditional metastasis (165).
- Metabolic reprogramming: Altered energy metabolism is one of the key cancer hallmarks, with the well-known example being the Warburg effect. The metabolic preferences vary when the tumor is in a pre-cancerous, *in situ*, or metastatic stage, due to continuous changes in the response to the cell state and environmental conditions (**Figure 3H**). *MYC* is one such oncogene with varying metabolic requirements, and its role in breast cancer is well established. It regulates the elevated expression of *SLC5A6* (vitamin transporter in cancerous cells) and facilitates the transport of vitamin B5 and its conversion to coenzyme A (149). This process alters the metabolic pathways, such as tricarboxylic acid (TCA) and fatty acid biosynthesis, ultimately promoting tumor progression. During metastatic evolution, cancer cells exhibit context-dependent metabolic switching, such as the processes of glycolytic metabolism or oxidative phosphorylation of lymph node metastasis or the reliance on fatty acid oxidation to balance the redox of latent brain metastatic cells (183). The metabolic plasticity of cancer is not restricted only to cancer cells but extends throughout the TME (184, 185), as seen in CAFs that secrete lactate, lung mesenchymal-derived cells that transfer lipids, suppressing the functioning of NK cells, and arginine-depleting collagen-producing TAMs, leading to the suppression of CD8^+^ T cell functions. More such examples are provided in **Table 1**.
- Distant metastasis and progression: Breast cancer metastasis is a complex, multicellular, and highly regulated process involving local invasion of primary tumor cells, epithelial-to-mesenchymal transition, invasion into the bloodstream, survival of tumor cells in the circulatory system, and colonization of secondary distant sites. Bone, lung, liver, and brain are the most frequent metastatic sites, and each sites possess different microenvironments to favor survival of metastasizing cancer cells **[****Figure 3I****]** (186, 187). They have different mechanisms to influence organ-specific tropism via different signaling pathways, such as the chemokine pathway CXCL12/CXCR4 (161), integrin-mediated adhesion (163), and changes in the ECM and tumor cell extracellular vesicles to establish a pre-metastatic microenvironment (164, 165). The role of interactions between tumor cells and components of the stroma, immune cells, or endothelial cells can influence the efficiency of metastasis, resulting in reduction, persistence, or progressive metastasis.

Dysregulation in the above-mentioned factors are associated with relapse and resistance to therapy (**Figures 3J, K**).

## 6 Diagnostic approaches

**Figure 4:**
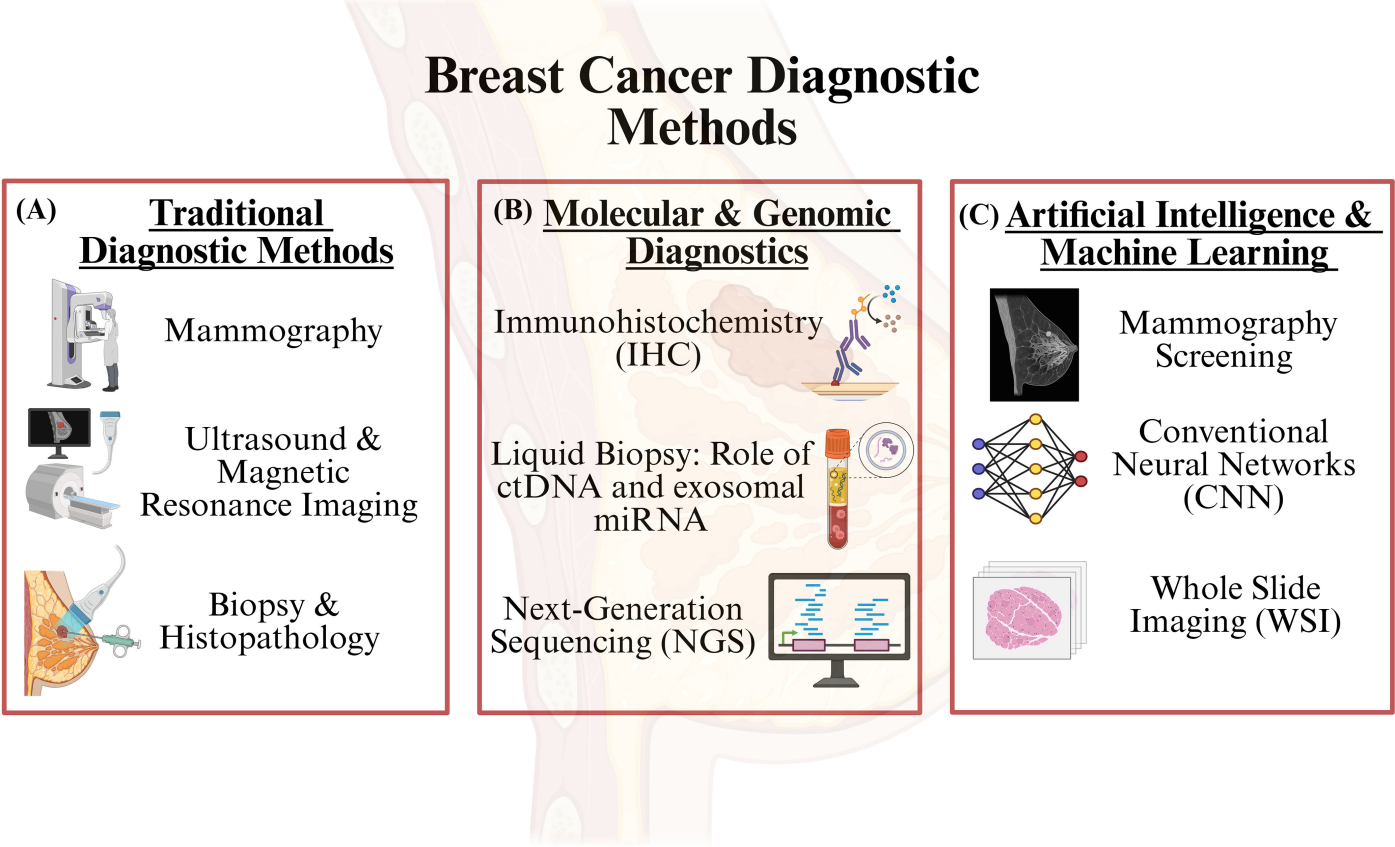
Breast cancer diagnostic methods. Breast cancer can be diagnosed via several methods. **(A)** In the first section, we present traditional diagnostic methods that have been implemented over the years, such as mammography, ultrasound, magnetic resonance imaging, and histopathology. **(B)** In the second section, we mention various molecular and genomic methods associated with diagnosis, including immunohistochemistry technique, liquid biopsy (circulating DNA, exosomal miRNA, lncRNA, etc.), and next-generation sequencing techniques. **(C)** Lastly, I have mentioned advanced artificial intelligence and machine learning-based diagnosis techniques, which include deep learning models developed using whole slide imaging for the diagnosis, AI-based models developed using mammography screening, and more. BioRender.com was used to create the diagram.

Breast cancer diagnosis has undergone significant transformation in the last one or two decades. It has advanced from classical imaging and histological evaluation to modern day molecular and genomic techniques. Both categories have their unique advantages and complement each other in clinical practice. For improved patient clinical outcomes, personalized therapies, and improved survival rates, accurate and early diagnosis is a prerequisite. Here, in this review, we have discussed both the traditional and modern techniques (**Figure 4**).

### 6.1 Traditional diagnostic methods

#### 6.1.1 Mammography

Until now, mammography continues to be the most effective method for screening and early detection of breast cancer. It uses the low dose of X-rays to detect masses, microcalcifications, and architectural distortions suggestive of malignancy. Its large-scale uses have reduced the mortality rate by facilitating earlier intervention before the tumor becomes palpable. Digital mammography has further enhanced the sensitivity and specificity compared to analog mammography, especially in women over the age of 50. However, the sensitivity reduces in younger women with denser breast tissue, resulting in false negatives and delayed diagnoses (188). All the aforementioned problems make the case for the need for auxiliary imaging modalities. Despite these problems, mammography remains the gold standard due to its accessibility, cost-effectiveness, and impact on public health.

#### 6.1.2 Ultrasound and magnetic resonance imaging: supplemental imaging techniques

Ultrasound imaging is another alternative to mammography, especially for those situations where there are dense breast tissues. Ultrasound imaging offers several advantages that include the ability to differentiate between cysts and solid masses, making biopsies easier, margins, blood vessels within tissues, and lymph nodes. This improves diagnostic accuracy (189). Another imaging test used is Magnetic Resonance Imaging (MRI), which is highly advanced and proven to be better than the other imaging tests. This technique is important for instances relating to high-risk screening, preoperative staging, and postoperative evaluation. In other instances, MRI has proven to increase its sensitivity level to 90%, making it highly effective compared to other techniques such as mammography (190). Unfortunately, MRI is costly, and lower specificity may result in false positives, highlighting the importance of selecting patients carefully. Together, ultrasound and MRI have addressed the mammography limitation and complement it by enhancing diagnostic accuracy.

#### 6.1.3 Biopsy and histopathology: definitive diagnosis

While imaging techniques elucidate important information on the disease’s presence and its extent, examination of tissue via a histopathological approach remains the definitive diagnosis method. The commonly used biopsy technique comprises fine needle aspiration (FNA), excisional biopsy, and core needle biopsy, with core needle being the preferred one because of its capability to preserve tissue architecture and facilitate receptor testing (191). Histopathological evaluation determines tumor type, its grade, and its histological subtype, guiding prognosis and suitable therapy.

### 6.2 Molecular and genomic diagnostics

#### 6.2.1 Immunohistochemistry

Immunohistochemistry represents an advanced, more complex tool in the diagnosis of breast cancer. The tool routinely investigates the expression of the receptors ER, PR, and HER2/neu in tissue biopsy and is applied later in tumor subtype classification. It also allows the detection of protein biomarkers within the tumor tissues (192). IHC has enabled researchers and clinicians to pinpoint prognostic markers and develop targeted therapy. The technique is usually standardized globally; although some variation can occur within individual laboratories, reflecting the importance of quality control and expert interpretation. More appropriate tumor characterization and prognosis have become possible with refinements in newer multiplex panel IHC over the last few years.

#### 6.2.2 Liquid biopsy: role of circulating tumor DNA and exosomal miRNAs

Liquid Biopsy is an advanced and emerging form of non-invasive and dynamic technique that detects and analyzes the biomarkers present in the bloodstream for the genotyping of an existing tumor. Circulating tumor DNA (ctDNA) comprises the tumor-derived DNA fragment released into the bloodstream, depicting the real-time tumor burden and molecular alterations (193). Liquid biopsies also enable the early detection of the recurrence of tumors, the assessment of minimum residual disease, and the evaluation of treatment responses without the need for repeated biopsies. Exosomal microRNAs (exomiRs), found within extracellular vesicles (EVs), represent a novel class of markers for the regulation of the growth and dissemination of breast cancer (194). ExomiRs offer promising areas for developing personalized management, though challenges persist regarding their sensitivity, standardization, and clinical validation.

#### 6.2.3 Next-generation sequencing

Recent advances in the field of next-generation sequencing (NGS) have completely revolutionized the field of cancer genomics. It has allowed researchers and clinicians to perform comprehensive profiling of tumor DNA to identify somatic mutations, copy number variations, cis and trans gene fusions, splicing events, and more. In the case of breast cancer as well, key mutations in genes such as *BRCA1, BRCA2, TP53, PIK3CA*, etc., influencing prognosis and treatment have been characterized. By identifying mutations in a patient-specific manner, NGS has enabled the design of guided therapies, predictive testing, or clinical trials (195, 196). With the advancements in single-cell and spatial transcriptomics, understanding the role of clonal evolution and tumor heterogeneity in drug resistance mechanisms is much easier (197). Despite challenges such as high cost and technical demands, NGS has been employed in clinical settings for routine diagnostics at several cancer centers.

### 6.3 Artificial intelligence and machine learning in diagnostic approaches

The role of AI technology and ML techniques is becoming integral in the field of breast cancer diagnosis. To begin with, the role of AI and ML technology in the field of mammography screening has been explored in many studies and trials, wherein the detection and diagnosis of breast cancer were done with the help of AI technology. In many studies, the use of AI in the screening process resulted in an increased detection rate of cancer in women and a reduced workload for radiologists as well as a reduction in the incidence of interval cancer in women. In fact, the MASAI randomized trial also proved the efficacy of the use of AI technology in the detection and diagnosis of breast cancer in women (198). Apart from screening, other techniques include convolutional neural networks (CNNs), a form of deep learning and radiomics-based ML algorithms for MRI, tomosynthesis, and ultrasound to predict treatment response, receptor status, or the chance of recurring through the use of quantifiable characteristics that recognize the heterogeneities of a tumor or the TME. Robust performance for MRI radiomics and a combination of radiomics and DL algorithms has been reported in several multicohort studies for the prediction of treatment response and molecular subtypes (199, 200).

In digital pathology, several studies employing DL algorithms using whole slide imaging (WSI) of H&E-stained tissue sections have demonstrated their power in predicting the receptors’ status, proliferation markers, tumor identification, and tumor grade, and even tumor-infiltrating lymphocytes. More recent studies have demonstrated the potential of DL algorithms in the quality aspects of pathology (201, 202). Commercially available AI-based products such as Lunit INSIGHT MMG and ProFound AI, have obtained FDA approvals and are in use to support mammographic image reading, particularly in cases where there is a workforce issue or when breast density is high. Prospective studies and randomized trials are currently underway to generate more sophisticated data for inclusion within the screening pathway (203).

Notably, methodological developments we have emphasized include transfer learning of pretrained models from large cohorts, multimodal models fusing imaging information with clinical and/or genomics, explainability techniques for the interpretation of models-e.g., saliency maps, Grad-CAM-and federated learning methods to address privacy and generalization issues across institutions. While the results show great promise, significant challenges remain in reducing algorithmic bias across demographic groups, validating performance with diverse external datasets, prospective clinical evaluations, standardization of performance metrics, and clear regulatory pathways towards deployment (203, 204).

## 7 Treatment strategies for breast cancer

The strategies for treating breast cancer have progressed in tandem with our understanding of the disease’s molecular and pathological heterogeneity, surpassing the notion of a universal approach. Current treatment includes radiation, chemotherapy, surgery, hormonal therapy, targeted therapies, and emerging immunotherapies. Appropriate treatment is decided based on tumor stage, grade, status of receptor, genetic mutations, patient’s age, overall health, and more. With the advancements in the personalized medicine approach, clinicians design treatment to maximize benefits and efficacy with minimum side effects and toxicity. Here, we have discussed these treatment strategies, exploring them from traditional to modern approaches.

### 7.1 Surgical interventions

Surgical intervention is broadly and routinely used in breast cancer treatment, involving removal of part or all of the breast. There are 3 different kinds of surgical interventions: (a) lumpectomy, (b) mastectomy, and (c) sentinel lymph node biopsy (SNLB) (205). Lumpectomy is a surgical technique that involves the removal of a tumor or lump along with some normal tissue surrounding that tumor. The technique is also termed “breast-conserving surgery” in which an attempt is made to preserve the maximum amount of tissue. The procedure is completed through radiation therapy to ensure minimum relapse. The technique of lumpectomy has an equal rate of survival in patients with early stages of breast cancer (206). Next is mastectomy, whereby one or both breasts are removed. It is used in cases where there are many growths in the breast, or the growth is bigger than the breast itself. Progress has been made in enhancing mastectomy techniques where the skin or nipples may be left behind to ensure cosmetic outcomes without compromising the oncological outcomes (207). Lastly, in the case of SNLB, the surgeon will remove a sentinel lymph node for examination of the metastatic condition, thereby minimizing the development of lymphedema compared to full axillary dissection (208).

### 7.2 Radiation therapy

Radiotherapy is of significant importance in the treatment of a patient having breast cancer as it reduces the recurrence risk of the disease by killing the cancerous cells post-surgery, particularly mastectomy (209). Although the patient experiences a couple of side effects after the treatment, the radiotherapy has a significant impact on the life expectancy of the patient with the disease. There are 2 kinds of treatments used in the radiotherapy of a patient with the disease; specifically, they are as follows: (a) EBRT and (b) Brachytherapy. EBRT is the most common method, in which higher levels of X-ray beams are directed to the specific area or the entire region of the breast. For enhancing the precision of the EBRT method, techniques like three-dimensional conformal radiation therapy (3D-CRT) and intensity-modulated radiation therapy (IMRT) with minimal exposure to the surrounding tissues have been developed (210). Next, there is brachytherapy, which works by inserting the radioactive sources in or near the tumor site, enabling a high dose in a short period of time. This modality is advantageous in the case of accelerated partial breast irradiation (APBI), as the dose is delivered to specific areas like the lumpectomy site itself (211).

### 7.3 Systemic therapies

Systemic therapy plays an important role in breast cancer as it helps combat the tumor cells far from the original site of the tumor, whether its early or late-stage condition. The benefits associated with systemic therapies for breast cancer involve reducing the rate of relapse, enhancing the survival rate in early stages, and inhibiting breast cancer from spreading to other internal organs (212). The different systemic therapy techniques for managing breast cancer involve hormone therapy, chemotherapy, targeted therapy, and immunotherapy, all of which have a different mechanism for treatment and a different outcome, reliant on various factors (tumor grade, receptor status, and molecular subtype). Below we have discussed these therapies in a summarized manner.

Hormone therapy is the preferred therapy for treating tumors that are HR positive with an aim to block the receptor action or suppress its production. One such example is tamoxifen, a selective estrogen receptor modulator (SERM), which prevents estrogen-driven cancer cell proliferation by binding to estrogen receptors and is particularly valuable for premenopausal women (213). According to a study done by the Early Breast Cancer Trialists’ Collaborative Group, 2005, tamoxifen has shown its potential by elevating the survival expectancy and reducing the relapse rate. Similarly, drugs from the class of aromatase inhibitors (AIs), including letrozole, anastrozole, and exemestane, have also shown positive results in postmenopausal women (214).

Chemotherapy, another form of systemic therapy, remains a cornerstone of breast cancer treatment, especially for aggressive subtypes such as TNBC or when the tumor is in an advanced stage and requires rapid cytoreduction. Anthracyclines such as doxorubicin and epirubicin cause cell death and cytotoxicity by intercalating into DNA and disrupting replication, this is part of standard treatment (67, 215). Similarly, drugs from the class taxanes, such as paclitaxel and docetaxel, complement anthracyclines by stabilizing microtubules and inhibiting cell proliferation; their sequential administration has shown improved efficacy of the treatment (216).

**Figure 5:**
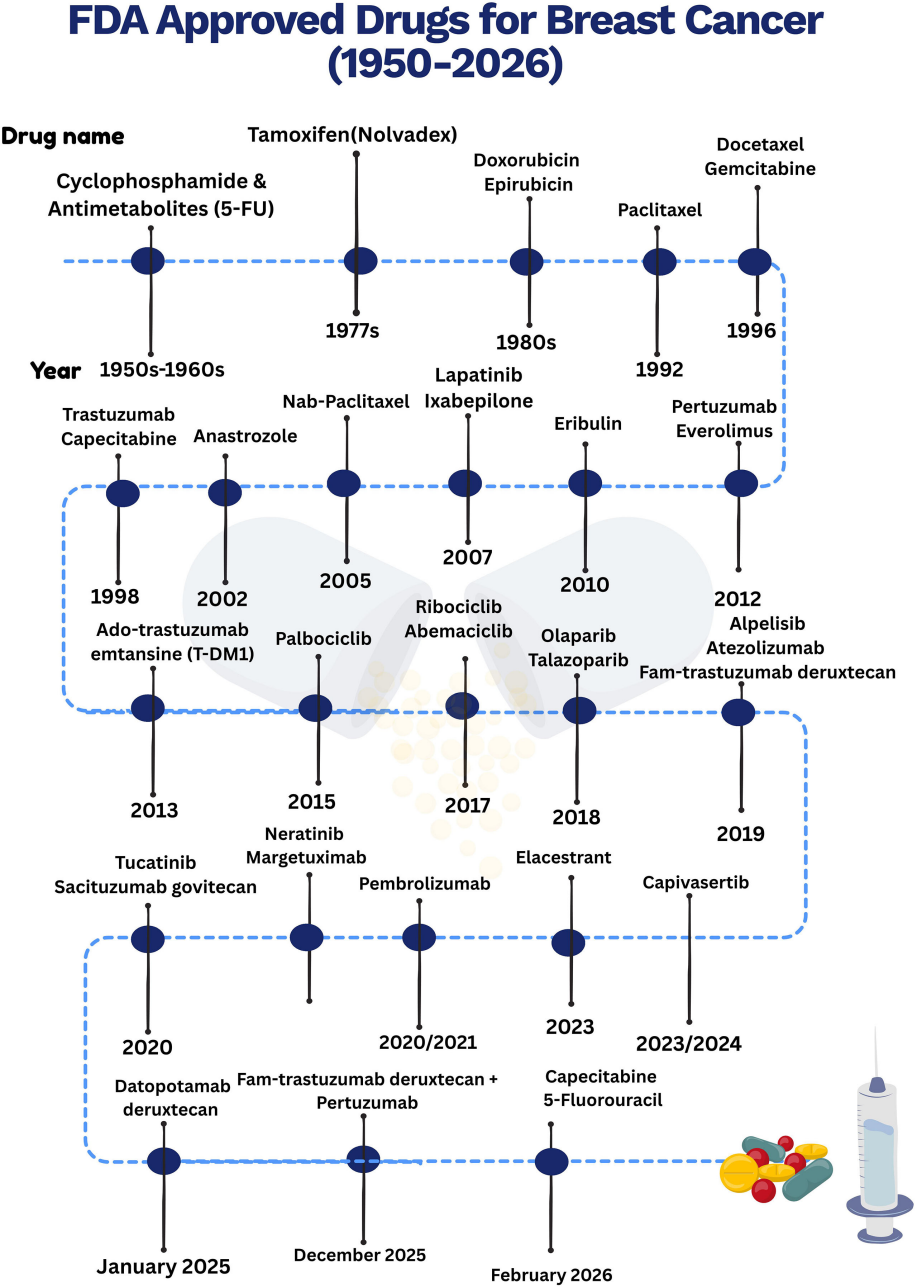
FDA approved drugs against breast cancer. In the provided figure we have compiled the drugs approved by FDA starting from year 1950s to the recent one’s i.e. 2026. These drugs belong to different classes i.e. small molecule-based chemotherapeutics, small molecule-based targeted therapies (kinase/mTOR inhibitors, etc.), monoclonal antibodies-based drugs, therapies targeting hormones, and more.

Targeted therapies are another popular form of systemic therapy, in which molecular drivers of tumor progression are selectively targeted. For instance, trastuzumab (a monoclonal antibody) is designed to target the HER2 receptor in the case of HER2-positive tumors, where it has demonstrated a significant improvement in survival rates (disease-free as well as overall survival) in early stage and metastatic disease settings (217). Another such example is of cyclin-dependent kinases 4 and 6 (CDK4/6) inhibitors (palbociclib, ribociclib, and abemaciclib) developed to target HR-positive and HER2-negative breast cancers. These drugs inhibit cell cycle progression at G1/S checkpoint and exhibit significant improvement in progression-free survival and overall survival (in some cases) when combined with endocrine therapy (218). Changes in late 2025 and early 2026 now include shifting Antibody-Drug Conjugates (ADCs) earlier in treatment. These shifts from the third or fourth line of care to the earlier stages of cancer care, now including first or second-line settings, are because they are very potent in chemotherapy without significant systemic side effects (219). The integration of these targeted therapy agents approved by the FDA into clinical practice is certainly a step ahead in precision medicine in the treatment of cancer **[****Figure 5****]**, for it enables the stratification of therapies according to tumor biology and optimizes treatment outcomes for unique types of breast cancer. **Table 2** provides list of ongoing clinical trials evaluating targeted therapy response against HR+ and HER2- locally advanced or metastatic breast cancer.

**Table 2:** Clinical trials against patients with HR+/HER2- locally advanced or metastatic breast cancer.

Therapy stage	Trial name	Molecular subtype	Agent	Target	Clinical phase	Sample size	Arms	Status	Reference
Prior Endocrine Therapy	SANDPIPER (NCT02340221)	ER+, HER2-, PIK3CA-mutant	Taselisib	PI3K	III	516	Tas + Ful *vs* Pcb + Ful	Not approved	(220)
Prior Endocrine Therapy	SOLAR (NCT02437318)	HR+, HER2-	Alpelisib	PI3K	III	341	Alp + Ful *vs* Pcb + Ful	Approved	(221, 222)
Prior Endocrine Therapy+CDK4/6i	BYLieve (NCT03056755)	HR+, HER2-	Alpelisib	PI3K	II	121	Alp + Ful	Approved	(223)
First Line	INAVO120 (NCT04191499)	PIK3CA-mutated, HR+, HER2-	Inavolisib	PI3K	III	325	Pal + Ful + Ina *vs* Pal + Ful + Pcb	Approved	(224)
Prior Endocrine Therapy	FAKTION (NCT01992952)	PIK3CA, AKT1, or PTEN	Capivasertib	AKT	III	140	Ful + Cap *vs* Pcb + Cap	Approved	(225, 226)
Prior Endocrine Therapy+CDK4/6i	CAPItello-291 (NCT04305496)	HR+, HER2-	Capivasertib	AKT	III	708	Cap + Ful *vs* Pcb + Ful	Approved	(227)
No prior chemotherapy for ABC or relapse >1 year of neoadjuvant chemotherapy	IPATunity130, cohort B (NCT03337724)	HR+, HER2-, HER2 metastatic breast cancer (aBC)	Ipatasertib	AKT	III	222	Ipa + Pac *vs* Pcb + Pac	Not approved	(228)
Prior Endocrine Therapy	TAMRAD (NCT01298713)	HR+, HER2-	Everolimus	mTOR	II	111	Eve + Tam *vs* Tam	Approved	(229)
Prior Endocrine Therapy	BOLERO-2 (NCT00863655)	HR+, HER2-	Everolimus	mTOR	III	724	Eve + Exe *vs* Pcb + Exe	Approved	(230, 231)
Prior Endocrine Therapy	PrE0102 (NCT01797120)	HR+, HER2-	Everolimus	mTOR	II	131	Ful + Eve *vs* Ful + Pcb	Not approved	(232)

Tas, Taselisib; Ful, Fulvestrant; Pcb, Placebo; Alp, Alpelisib; Pal, Palbociclib; Ina, Inavolisib; Cap, Capivasertib; Ipa, Ipatasertib; Pac, Paclitaxel; Eve, Everolimus; Tam, Tamoxifen; Exe, Exemestane.

In this table, we have compiled all the clinical trials (completed and ongoing) against patients with HR+/HER2- locally advanced or metastatic breast cancer. For each clinical trial, we have enlisted the therapy stage, name of the trial, molecular subtype, agent and their target, sample size, the clinical phase they are in, approval status and the arms, i.e., the manner in which they are given to patients.

Immunotherapy is another evolving class of systemic therapy that has gained attention in the last few years for treating carcinomas, particularly for the TNBC subtype, which lacks treatment options because of receptor absence. The most classic example is immune checkpoint inhibitors (ICIs) (pembrolizumab and atezolizumab), which target the PD-1/PD-L1 axis and have shown clinical benefits by reactivating anti-tumor T-cell responses. Treatment efficacy increases, particularly in PD-L1-positive tumors, offering a new hope for TNBC patients (233). **Table 3** provides list of ongoing clinical trials evaluating ICIs response against TNBC.

**Table 3:** Key ICIs based clinical trials against TNBC.

Therapy stage	Trial name	Molecular subtype	Agent	Clinical phase	Sample size	Arms	Status	Reference
First Line of Advanced Breast Cancer	IMpassion130 (NCT02425891)	TNBC	Atezolizumab	III	902	Nab-Pac+ Atz *vs*. Pcb+ Atz	Approved	(234, 235)
	IMpassion131 (NCT03125902)	TNBC	Atezolizumab	III	651	Pac+ Atz *vs* Pcb+ Atz	Not-Approved	(236)
	KEYNOTE-355 (NCT02819518)	TNBC	Pembrolizumab	III	847	Chm+ Pmb *vs* Chm+Pcb	Approved	(237, 238)
	TORCHLIGHT (Not Available)	TNBC	Toripalimab	III	531	Nab-Pac+Tpb *vs* Pcb+ Nab-Pac	Approved	(239)
Neoadjuvant Systemic Treatment	I-SPY2 (NCT01042379)	TNBC, HR+/HER2-Negative	Pembrolizumab	II	114	Pac+ Pmb	Approved	(240)
	GeparNuevo (NCT02685059)	TNBC	Durvalumab	II	174	Nab-Pac+dmb *vs* Nab-Pac+Pcb	Approved	(241, 242)
	KEYNOTE-522 (NCT03036488)	TNBC	Pembrolizumab	III	1174	Chm+ Pmb *vs* Pcb+ Pmb	Approved	(243–245)
	Impassion301 (NCT01633970)	TNBC	Atezolizumab	III	313	Nab-Pac+ Atz *vs* Nab-Pac+Pcb	Approved	(246)
	NeoTRIPaPDL1 (NCT02620280)	TNBC	Atezolizumab	III	280	Nab-Pac+Cpt± Atz	Not-Approved	(247)
Adjuvant Systemic Therapy	Impassion030 (NCT03125902)	TNBC	Atezolizumab	III	2199	Chm+ Atz *vs* Chm	Not-Approved	(248)

Atz, Atezolizumab; Pmb, Pembrolizumab; Tpb, Toripalimab; Dmb, Durvalumab; Chm, Chemotherapy; Cpt, Carboplatin; Pac, Paclitaxel; Pcb, Placebo; TNBC, Triple Negative Breast Cancer.

In this table, we have compiled all the clinical trials (completed and ongoing) against TNBC patients. For each clinical trial, we have enlisted the therapy stage, name of the trial, molecular subtype, agent, sample size, the clinical phase they are in, approval status, and the arms, i.e., the manner in which they are given to patients.

### 7.4 Machine learning guided therapeutic decision making

Currently, machine learning (ML) techniques are being increasingly employed for better optimization and treatment of breast cancer through prediction, therapy response, drug sensitivity, resistance, and patient-specific outcomes. ML have already shown promising results utilizing multiomics-based models for several therapies including chemotherapy, endocrine therapy, targeted therapy, and immunotherapy response forecasting (249, 250). Several radiomics-based ML models that combine MRI and clinical features can precisely predict pathological complete response to neoadjuvant chemotherapy, and thus inform timely modifications to therapy and individualized therapy design (251). Another commonly utilized ML framework for predicting drug response in breast cancers relates to identifying patterns of drug sensitivity and resistance via gene-expression signatures, aiding individualized therapy decisions (252, 253).

Network-based bioinformatics, integrated with ML, has also eased the repurposing of drugs as well as the identification of synergistic combinations, based on the match between tumor molecular signatures and the pharmacogenomic data from GDSC and CCLE (254). In HR+ and HER2+ breast cancers, ML models have established the ability to correctly classify patients for CDK4/6, HER2, and PI3K pathway inhibitors, which is beneficial for precision oncology (255). Additionally, the utility of ML was made possible through the application of ML in digital pathology and TME profiling, based on which it was possible to predict the response to immunotherapy based on the quantification of tumor-infiltrating lymphocytes and immune-related features (256). Thus, therapeutic modeling using ML has been found to be instrumental in the treatment of breast cancer using individualized treatment modalities.

### 7.5 Plant-based therapy and traditional knowledge

Plant-based drugs have always been an essential component of anticancer drug discovery, as they serve as natural reservoirs of compounds with immense anticancer properties. Most of the anticancer drugs being used today have been derived from plants and have been developed into effective drugs. In breast cancer, phytochemicals have been used both as chemotherapeutic agents and as adjuvants to improve the response to treatment (257).

Plant-based drugs sourced from international countries (other than India) have been the primary source of many current standard treatments for breast cancer. Paclitaxel (Taxol), which was first derived from the Pacific yew tree, is still one of the mainstay drugs that target microtubule function and inhibit tumor cell division (258). Vinca alkaloids like vinblastine and vincristine, which were first obtained from the Madagascar periwinkle plant, are mitosis inhibitors and have been used for many years in cancer chemotherapy (259). Another significant class of drugs consists of camptothecin analogs such as irinotecan, which are derived from the plant Camptotheca acuminata and function as topoisomerase I inhibitors. These drugs have been shown to possess anticancer activity in different cancers, including breast cancer (260).

Conversely, Indian plant-based medicines combine both globally harnessed chemotherapeutic agents and traditional Indian systems of medicine, making a significant contribution to breast cancer treatment and management. India is a breeding ground for the global production of periwinkle-based vinca alkaloids and Himalayan yew-based taxanes. Indigenous medicinal plants like Ashwagandha (withaferin A) trigger apoptosis and radiosensitization in aggressive breast cancer cells (261, 262), while curcumin from turmeric inhibits NF-κB signaling pathways and increases the effectiveness of chemotherapy (263). Other plant-derived compounds such as piperine target drug resistance and oncogenic signaling such as HER2 (264). Ayurvedic drugs such as Triphala (265), Panchakola (266), Maharishi Amrit Kalash (267), and newly developed nano-Ayurvedic drugs such as Nano Swarna Bhasma function as cytotoxic agents and adjuvants that reduce the toxic effects of chemotherapy and also potentially limit metastasis (268). Therefore, plant-derived medicines from India offer a complementary strategy that leverages both the direct and potentiating anticancer activities.

## 8 Bioinformatics and AI in breast cancer research

### 8.1 Role of bioinformatics

In the past few decades, advances in the area of bioinformatics have smoothly transformed cancer biology research, particularly in breast cancer research, by integrating a large volume of biological data with computational approaches to identify new information that was previously inaccessible by traditional experimental methods alone. It has facilitated researchers and data analysts to integrate multi-omics data to generate new ideas and hypotheses, discover new diagnostic and prognostic biomarkers and therapeutic targets, perform disease stratification, and more. Breast cancer research has become more data driven due to advancements in computational power and algorithmic sophistication. It facilitates clinicians and researchers strategizing therapeutic interventions based on molecular signatures rather than histopathology data alone. Below we have discussed these key factors in detail.

#### 8.1.1 Genomic and system-level approaches

In the last two to three decades, NGS technologies have generated large amounts of omics data (genomics, transcriptomics, proteomics, epigenomics, and metabolomics), post-translational modification data (phosphorylation, SUMOylation, ubiquitinylation, etc.), single-cell, ATAC-Seq data and more, which provide a powerful lens for deciphering the molecular landscape of breast cancer (269). Large-scale projects such as The Cancer Genome Atlas (TCGA) (270), Molecular Taxonomy of Breast Cancer International Consortium (METABRIC) (271), International cancer genome Consortium (ICGC) (272), Pan Cancer Analysis of Whole Genome (PCAWG) (273), National Cancer Institute-Clinical Proteomic Tumor Analysis Consortium (NCI-CPTAC) (274), and Memorial Sloan Kettering-Integrated Mutation Profiling of Actionable Cancer Targets (MSK-IMPACT) (275) have generated high-resolution datasets that include bulk gene expression data, somatic mutations, Copy Number Variations (CNVs), methylation patterns, cell-type-specific gene expression profiles, acetylation, and phosphorylation across thousands of samples collected from diverse races and geographical locations. These resources have led to refinement of BC classification in four molecular subtypes, i.e., Luminal A, Luminal B, HER2, and Basal. For example, a comprehensive analysis of TCGA-BRCA data showed recurrent mutations/variations were detected in genes such as *TP53, PIK3CA, GATA3*, and *MAP3K1*, associated with tumor initiation, progression, and therapy resistance (276). Beyond mutation profiling, genomic data enables characterization of predictive, prognostic, and diagnostic biomarkers and new therapeutic targets based on differential gene expression studies. Analytical methods such as principal component analysis (PCA) and machine learning (supervised and unsupervised) stratify tumors into aggressive and non-aggressive and decipher novel biological insights (277). Further, genomic studies have enabled the design of personal gene panels and decision tools that permit the estimation of the probability of recurrence and treatment strategies, such as Oncotype DX (278) and MammaPrint (279). Furthermore, with the improvements in the newer technologies, such as cloud-based, researchers and clinicians are more motivated to integrate genome profiles with unprecedented depth and precision for improving data-driven patient outcomes.

#### 8.1.2 Pathway enrichment analysis

Genomics analysis is complemented by pathway enrichment analysis, where the focus is shifted from single gene alteration to biological system-level alteration. It provides a better understanding of the intricate molecular mechanism responsible for driving carcinogenesis. The information regarding enriched pathways or processes is collected from in silico resources such as Database for Annotation, Visualization and Integrated Discovery (DAVID) (280), Kyoto Encyclopedia of Genes and Genomes (KEGG) (281, 282), Enrichr (283), etc. by providing a gene signature as an input. These gene signatures are then mapped to the list of curated biological pathways, revealing key dysregulated processes. For example, gene set enrichment analysis (GSEA) using DEGs from the HER2 subtype has revealed enrichment of processes such as “HER2 signaling cascades,” linking with their clinical behavior and response to targeted therapy (284). Likewise, in the case of TNBC, immune-related processes such as “interferon signaling” and “antigen presentation” have been observed (285), laying the groundwork for ongoing immunotherapy. Moreover, pathway-based strategies have enhanced the multi-omics data interpretation into a unified biological context, improving biomarker discovery and uncovering hidden regulatory networks, which are often missed in single data type-based studies. Advanced tools such as Enrichr (283), Metascape (286), etc. allow visualizing multi-omics data, making complex data easier to interpret in clinically relevant hypotheses.

#### 8.1.3 Network biology approach

While pathway analysis highlights the dysregulated processes, network-based approaches extend this by mapping the interaction between genes, proteins, and molecular regulators within complex cellular systems, offering a complete holistic view of system-level changes (287, 288). Network biology implemented computational models to interpret gene regulatory networks (GRNs) (289), protein-protein interaction (PPI) networks (290) and signaling cascades (291), characterizing the key modules, hubs, and bottlenecks linked with cancer phenotypes. In breast cancer research, network tools such as Cytoscape (292), STRING (293), and Weighted Gene Co-expression Network Analysis (WGCNA) (294) have characterized several functional modules, such as the PD-L1/PD-1 axis, cyclin-dependent kinases (CDKs), BRCA1/2 complexes, and more, by analyzing transcriptomics and proteomics together. In another study, the network analysis projected ErbB2 consistently as a central node in the HER2 subtype, interacting with proteins PI3K, SHC, and GRB2. This finding underscores the role of ErbB2 as a key pathogenesis driver and molecular target for targeted therapies (295). In addition, network statistical matrices such as degree centrality, betweenness, or clustering coefficients facilitate prioritizing key genes for experimental validation, especially in the case of TNBC, where there are very few druggable targets (296). Network biology’s potential lies in its ability to integrate diverse data types into meaningful biological concepts, allowing researchers to understand the tumor characteristics missed by linear pathway analysis. This system-level approach enables combinatorial and rational drug designing and characterization of new master regulators for precision medicine.

### 8.2 AI in diagnosis and prognosis: transforming clinical precision with algorithms

Recent advances in the last decade in artificial intelligence have completely transformed the breast cancer diagnosis and prognosis. It has allowed researchers and clinicians to interpret medical data and predict outcomes more accurately. By leveraging a large volume of health records and slides from digital pathology datasets, AI-based models, particularly deep learning-based models, detect subtle events that go unnoticed by the human eye (297). The big revolution has come in the field of imaging, where convolutional neural networks have either matched or outperformed radiologists in mammogram interpretation. Models have been developed to predict gene expression, prognosis, and more on several image datasets such as INbreast (298), Curated Breast Imaging Subset of Digital Database for Screening Mammography (CBIS-DDSM) (299), and more. CNNs detected asymmetries, microcalcifications, and architectural distortions with high sensitivity, even in dense breast tissue, an area where even radiologists often fail (300). AI is not just limited to imaging; methods like natural language processing (NLP) are now being used to gather important information from messy clinical reports such as the status of hormone receptors, health situation, treatment provided in the past, and more. Furthermore, AI tools such as DeepSurv (301) and Cox-nnet v2.0 (302) facilitate the development of personalized survival prediction by integrating genomic, clinical, and histopathological data. They have been shown to outperform traditional tools in several cohorts. In resource-limited environments, AI can function as a decision support system, for example. Google’s tool LYmph Node Assistant (303) (LYNA) can detect metastases in pathological slides with high accuracy, facilitating pathologists triaging difficult cases. AI tools, in addition, have been developed to predict therapy responses (pathological complete response (pCR), neoadjuvant chemotherapy) based on treatment MRI scans and molecular profiles (304). Such innovations save time in diagnosis and enable precision medicine by tailoring therapies to individual tumor biology. Despite the advantages, the application of AI is challenged in many facets, including the “black box” effect of DL models, bias in the datasets used for the model, and the models’ ability to be generally applicable to the population and imaging modalities used. Nevertheless, the use of AI technology in association with the field of molecular oncology offers a paradigm shift for the creation of diagnostic and prognostic tools that are fast, smart, and hold the potential for better patient outcome (305).

### 8.3 AI in drug discovery and personalized therapy

AI is transforming the field of drug development, particularly in breast cancer, by elevating the novel therapeutics discovery and promoting personalized treatment. By integrating and analyzing multi-omics data together, AI enables the discovery of novel biomarkers and new druggable targets, performs drug repurposing, and saves both cost and time with respect to conventional methods (306). AI has shown tremendous performance in structure-based drug discovery because of its ability to develop robust ML and DL models on structural and biochemical data to predict protein-ligand interactions, outperforming conventional high-throughput screening with high efficiency and accuracy. For example, AI-enabled virtual screening characterizes novel inhibitors for breast cancer targets such as ER and HER2. Classic examples of two such widely used AI-driven platforms include DeepChem (307) and AtomNet (308). By integrating AI with a library of compounds and multi-layered breast cancer datasets, it will facilitate refining candidate selection as per tumor subtype, receptor status, and molecular alterations.

List of all such bioinformatics and AI based tools and resources specific to breast cancer research is provided in **Table 4**.

**Table 4:** Computational resources in breast cancer research.

Type	Name	Link	Description	Reference
Genomics or Proteogenomics	TCGA-BRCA	https://portal.gdc.cancer.gov/projects/TCGA-BRCA	Provide multi-omics datasets related to breast cancer	(270)
	METABRIC	https://ega-archive.org/studies/EGAS00000000083	Provide copy number and gene expression data for ~2000 breast cancer samples	(271)
	ICGC-ARGO	https://www.icgc-argo.org/	Provides genomics, epigenomics and transcriptomics data	(272)
	PCAWG	https://xenabrowser.net/datapages/?hub=https://pcawg.xenahubs.net:443	Provides catalog of genomics, epigenomics and transcriptomics data	(273)
	NCI-CPTAC	https://wiki.cancerimagingarchive.net/pages/viewpage.action?pageId=70227748	Provides multi-omics data including post-translational modifications from the same tissue sample	(274)
	MSK-IMPACT	https://www.mskcc.org/msk-impact	Genetic sequencing test to identify mutation in breast cancer	(275)
	cBioPortal	https://www.cbioportal.org/	Platform for visualizing, analyzing and downloading cancer data	(309)
	LinkedOmics	https://www.linkedomics.org/#/	Platform provides multiomics and clinical data for several cancer	(310)
	GTEx	https://www.gtexportal.org/home/	Provides tissue specific whole genome, transcriptome and loci data	(311)
Web-based Analysis and Exploration tool	UCSC Xena Browser	https://xenabrowser.net/datapages/?cohort=TCGA%20Breast%20Cancer%20	Platform to integrate and explore functional genomic data sets	(312)
	TIMER 2.0	http://cistrome.org/TIMER/	Web-based resource for evaluating clinical impact of immune cells in diverse cancer.	(313)
	KMplot (Breast)	https://kmplot.com/analysis/index.php?p=service	Web-based tool to perform survival analysis	(314)
	bc-GenExMiner	https://bio.tools/bc-genexminer	Web-based tool for comparing gene expression, prognostic analysis, etc.	(315)
	DAVID	https://davidbioinformatics.nih.gov/	Web-based functional annotation tool to understand biological processes using gene sets.	(280)
	Enrichr	https://maayanlab.cloud/Enrichr/	Web-based functional annotation tool to understand biological processes, pathways, and more using gene sets.	(283)
	KEGG	https://www.genome.jp/kegg/	Web-based resource providing information of high-level functions, and biological system.	(281, 282)
	Metascape	https://metascape.org/gp/index.html#/main/step1	Web-based functional annotation tool to understand common and unique pathways and protein networks	(286)
	GEPIA 2.0	http://gepia2.cancer-pku.cn/	Web-based tool to perform several gene-based analyses such as survival analysis, differential expression, etc.	(316)
	STRING	https://string-db.org/	Web-based tool for protein-protein interaction networks	(293)
R/Python/GitHub Packages	genefu	https://www.bioconductor.org/packages/release/bioc/html/genefu.html	R package providing collection of bioinformatics algorithms and gene signatures for molecular subtyping and prognostic analysis in breast cancer	(317)
	DeepSurv	https://github.com/jaredleekatzman/DeepSurv	Deep learning-based package for performing survival analysis.	(301)
	Cox-nnet v2.0	https://github.com/lanagarmire/Cox-nnet-v2.0	Neural network-based package for performing survival analysis, extended to large scale electronic medical records data	(302)
AI/ML/DL based resources	LYNA	NA	Deep Learning package for detecting metastatic breast cancer	(303)
	OncoLLM	https://www.triomics.com/how-it-works	A large language model trained on clinical data, guidelines and oncology textbooks, EHRs, to identify potential trial matches for clinical patients	(318)
Imaging Datasets	INbreast	https://www.kaggle.com/datasets/ramanathansp20/inbreast-dataset	A database of full-field digital mammography from breast cancer patients	(298)
	CBIS-DDSM	https://www.kaggle.com/datasets/orvile/cbis-ddsm-dataset/data	A database of 2620 curated scanned film mammography studies.	(299)
	VinDr-Mammo	https://vindr.ai/datasets/mammo	A multi-scale database of benchmark dataset for computer-aided diagnosis in full-field digital mammography	(319)
	BreakHis	https://www.kaggle.com/datasets/ambarish/breakhis	A database of 2480 benign and 5429 malignant breast cancer histopathological image.	(320)
	BACH	https://www.kaggle.com/datasets/truthisneverlinear/bach-breast-cancer-histology-images	A database composing of microscopy as well as whole-slide images of breast cancer.	(321)
	CAMEYLON16/17	https://camelyon16.grand-challenge.org/	A database of breast cancer hematoxylin and eosin stained whole slide image of lymph node section.	(322)

Here we have enlisted all the computational resources widely used in breast cancer research. We have enlisted all the databases, machine learning/deep learning-based tools and packages, and the imaging datasets. A link to each tool, along with its description, is also provided.

## 9 Conclusions

Breast cancer until today remains a significant global health threat characterized by multifaceted and complex etiology and diverse clinical presentation. This comprehensive review examines the evolving dynamic landscape of breast cancer research, pointing out the complexity of the disease and strategies implemented to tackle it. Firstly, we discussed the epidemiology and various risk factors such as genetic mutations (*BRCA1/2, TP53, PTEN*), hormonal influences, several lifestyle risk factors (obesity, smoking, etc.), and environmental factors (chemical exposure, air pollutants, etc.). Furthermore, the role of interplay between genetic predisposition and external factors is well-established in breast cancer development and progression. These key factors together or individually shape the disease risk, progression, and therapeutic response, underscoring the importance and need of a comprehensive understanding of the disease etiology. Next, we reviewed the four molecular subtypes, pathophysiology, and role of tumor microenvironment in breast cancer diagnosis, prognosis, and therapeutic response. We also discussed various mechanisms driving breast cancer progression, highlighting recent research and new insights.

Moving ahead, we discussed traditional and modern diagnostic approaches such as mammography, improved imaging, molecular diagnostics, tissue biopsy, and liquid biopsy (exomiRs, ctDNA), and how they have significantly improved the accuracy and sensitivity of the diagnosis and reduced false positive cases. These advancements help optimizing treatment strategies and ensure proper and timely care. Recent advances have completely transformed the way breast cancer is treated and continue to evolve at an unprecedented pace focused on proportionate reduction of overtreatment and developing more precision and personalized treatment with few minimal side effects within clinics. Newer and emerging therapies including plant derived compounds offer hope for an improved lifestyle and better chances for survival. Additionally, the importance of the quality of preserved human lives and the long-term care of breast cancer patients receives due emphasis, combating the challenges pertaining to treatment complications and mental health effects, as well as recurrence in the interest of improving the survival rate. Heterogeneity of breast cancers has been an issue and challenges the one size fits all concept, calling for the concept of precision medicine.

Advances in AI, bioinformatics, and computational methods have brought about a paradigm shift in breast cancer research. From genomic modeling to pathway modeling to network biology in breast cancer, AI-based biomarker research from pathological slides, prognostic research, target screening, drug repurposing in drug discovery research in breast cancer, computational methods have completely changed the research in breast cancer. Integration of multi-omics data facilitates a comprehensive system-level understanding of carcinogenesis and therapeutic vulnerabilities, which single-omics data fails to provide. These advancements are taking the field ahead towards personalized oncology, where provided therapies will be as per the patient’s molecular profile.

To reduce the morbidity and mortality rates associated with breast cancer, It is very important to apply the advancements being recorded in breast cancer research to daily-life practices. Regular breast self-examination from young adulthood onwards, clinical breast examination once every 1–3 years for women aged 20–39 years, and annual mammographic screening from ages 40–50 and beyond may increase detection rates (323). Recently, blood-based biomarkers including ctDNA, CTCs, and miRNA signatures have emerged as promising non-invasive tools for early diagnosis, minimal residual disease monitoring, and recurrence prediction (324). Furthermore, changes in lifestyle modifications including healthy body weight, breastfeeding, limiting alcohol intake, and regular physical activity reduces breast cancer risk (325). Collectively, integrating preventive awareness, early screening strategies, and new biomarker technologies can significantly reduce the fear of breast cancer by allowing early intervention and improved survival.

Despite major improvements in our knowledge of the biology of breast cancer and progresses in treatment, several critical challenges and research gaps still exists. First, the issue of intratumoral heterogeneity as well as tumor evolution is still a big challenge, underscoring the requirement for longitudinal multi-omics profiling and analysis to elucidate dynamic resistance mechanism (326). Second, the underlying molecular processes behind the occurrence of distant metastasis, dormancy, and late-stage disease recurrence, especially in hormone receptor-positive breast cancer, are not fully understood, making it difficult to develop efficient metastasis-preventive therapies (327). Third, while emerging advanced diagnosis tools such as liquid biopsy and AI-based imaging platforms show tremendous promise, the practical application of these technologies in the clinic is also limited by their prospective validation, standardization, and accessibility (328). Fourth, predictive biomarkers capable of reliably stratifying patients for immunotherapy and targeted treatments remain suboptimal, contributing to treatment variability and overtreatment in many cases (329). Finally, the integration of multi-omics datasets with the development of associated computational and functional validation platforms poses a technological challenge, for which systems-based approaches in the development of precision therapy and precision medicine are required (330). In addition, increasing awareness among the public against increasing incidences of breast cancer; understanding the role of TME in forming pre-metastatic niches and metastasis; reducing dataset bias or missing values while developing AI/DL models; interpretability and generalizability of AI models; and filling the gap between basic and translational research. Also, there is a need to establish collaborative platforms between interdisciplinary teams, including basic researchers, clinicians, and industry partners, to take bench work to a product or treatment.

In conclusion, breast cancer, being one of the most common cancers in women, remains the subject of scientific and clinical research, requiring more focused research for better understanding, prevention, and treatment of breast cancer.

## Glossary

3D-CRT: Three-dimensional conformal radiation therapy
AI: Artificial intelligence
AIs: Aromatase inhibitors
Anti-CTLA4: Anti-cytotoxic T-lymphocyte-associated antigen 4 antibody
Anti-PD1: Anti-programmed cell death protein 1
APBI: Accelerated partial breast irradiation
ATM: Ataxia-telangiectasia mutated gene
BC: Breast cancer
BMX/STAT3: Bone marrow and X-linked nonreceptor tyrosine kinase/Signal transducer and activator of transcription 3 protein
BPA: Bisphenol A
BRCA1: Breast cancer gene 1
BRCA2: Breast cancer gene 2
CAF: Cancer-associated fibroblasts
CDH1: Cadherin 1
CDK4/6: Cyclin-dependent kinases 4 and 6
CHEK2: Checkpoint kinase 2
CNN: Convolution neural network
CNV: Copy number variation
CR: Circadian rhythm
CSC: Cancer stem cells
CTC: Circulating tumor cell
ctDNA: Circular tumor DNA
CTL: Cytotoxic T Lymphocyte
DAVID: Database for annotation, visualization and integrated discovery
DDR1-mediated STAT1: Discoidin domain receptor 1-mediated Signal transducer and activator of transcription 1
DDT: Dichlorodiphenyltrichloroethane
DL: Deep learning
dsDNA: Double-stranded DNA
DTC: Disseminated tumor cell
EBRT: External beam radiation therapy
ECM: Extra cellular matrix
EDC: Endocrine disrupting chemicals
EGF: Epidermal growth factor
EMT: Epithelial-mesenchymal transition
ER: Estrogen receptor
ER+: Estrogen recptor positive
EV: extracellular vesicle
exomiRs: Exosomal microRNAs
FAT1: Fat atypical cadherin 1
FNA: Fine needle aspiration
GPX4: Glutathione peroxidase 4
GRB2: Growth factor receptor-bound protein 2
GRN: Gene regulatory network
GSEA: Gene set enrichment analysis
GWAS: Genome-wide association studies
HER2: Human epidermal growth factor receptor
HIF-1α: Hypoxia inducible factor-1α
HRT: Hormone replacement therapy
ICGC: International cancer genome consortium
ICI: Immune checkpoint inhibitors
IGF-1: Insulin-like growth factor-1
IHC: Immunohistochemistry
IMRT: Intensity-modulated radiation therapy
KEGG: Kyoto encyclopedia of genes and genomes
KK-LC-1: Kita-Kyushu lung cancer antigen-1
LAR: Luminal androgen receptor
LOX: Lysyl oxidases
LYNA: Lymph node assistant
MAPK: Mitogen-activated protein kinase
MDSC: Myeloid-derived suppressor cell
METABRIC: Molecular taxonomy of breast cancer
MHC: Major histocompatibility complex
ML: Machine learning
MMP: Matrix metalloproteinases
MRI: Magnetic resonance imaging
MSK-IMPACT: Memorial sloan kettering-integrated mutation profiling of actionable cancer
myCAF: Myofibroblastic CAF
NCI-CPTAC: National cancer institute-clinical proteomic tumor analysis consortium
NF-κB: Nuclear factor-kappa B
NGS: Next-generation sequencing
NK: Natural killer
NLP: Natural language processing
NO2: Nitrogen dioxide
PAH: Polycyclic aromatic hydrocarbons
PALB2: Partner and localizer of BRCA2
PARP: Poly ADP ribose polymerase
PCA: Principal component analysis
PCAWG: Pan-cancer analysis of whole genome
pCR: Pathological complete response
PDGF: Platelet-derived growth factor
PD-L1/PD: Programmed death ligand 1/Programmed cell-death protein 1
PFAS: Polyfluoroalkyl substances
PI3K/AKT: Phosphatidylinositol 3-kinase/protein kinase B
PLCγ: Phospholipase gamma
PM: Particulate matter
POP: Persistent organic pollutants
PR: Progesterone receptor
PTEN: Phosphatase and tensin homolog
RANK: Receptor activator of nuclear
RCD: Regulated cell death
ROS: Reactive oxygen species
SASP: Senescence-associated secretory phenotype
SERM: Selective estrogen receptor modulator
SHC: Src homolog and collagen homolog
SNLB: Sentinel lymph node biopsy
STAT3: Signal transducer and activator of transcription 3
STK11: Serine/threonine kinase 1
STRING: Search tool for the retrieval of interacting genes/proteins
TAM: Tumor-associated macrophages
TCA: Tricarboxylic acid
TCGA: The cancer genome atlas
TGF-β: Transforming growth factor-beta
TIL: Tumor-infiltrating lymphocytes
TMAO: Trimethylamine N-oxide
TME: Tumer microenvironment
TNBC: Triple-negative breast cancer
TNF-α: Tumor necrosis factor-alpha
TP53: Tumor protein p53
Tregs: Regulatory T cells
TSG: Tumor suppressor genes
TSPAN8: Tetraspanin-8
VEGF: Vascular endothelial growth factor
WGCNA: Weighted gene co-expression network analysis
α5β1-FAK/Src: Integrin α5β1 and Focal adhesion kinase/c-Src proto-oncogene.
